# Multiomics in primary and metastatic breast tumors from the AURORA US network finds microenvironment and epigenetic drivers of metastasis

**DOI:** 10.1038/s43018-022-00491-x

**Published:** 2022-12-30

**Authors:** Susana Garcia-Recio, Toshinori Hinoue, Gregory L. Wheeler, Benjamin J. Kelly, Ana C. Garrido-Castro, Tomas Pascual, Aguirre A. De Cubas, Youli Xia, Brooke M. Felsheim, Marni B. McClure, Andrei Rajkovic, Ezgi Karaesmen, Markia A. Smith, Cheng Fan, Paula I. Gonzalez Ericsson, Melinda E. Sanders, Chad J. Creighton, Jay Bowen, Kristen Leraas, Robyn T. Burns, Sara Coppens, Amy Wheless, Salma Rezk, Amy L. Garrett, Joel S. Parker, Kelly K. Foy, Hui Shen, Ben H. Park, Ian Krop, Carey Anders, Julie Gastier-Foster, Mothaffar F. Rimawi, Rita Nanda, Nancy U. Lin, Claudine Isaacs, P. Kelly Marcom, Anna Maria Storniolo, Fergus J. Couch, Uma Chandran, Michael Davis, Jonathan Silverstein, Alexander Ropelewski, Minetta C. Liu, Susan G. Hilsenbeck, Larry Norton, Andrea L. Richardson, W. Fraser Symmans, Antonio C. Wolff, Nancy E. Davidson, Lisa A. Carey, Adrian V. Lee, Justin M. Balko, Katherine A. Hoadley, Peter W. Laird, Elaine R. Mardis, Tari A. King, AURORA US Network, Aguirre A. De Cubas, Charles M. Perou

**Affiliations:** 1 University of North Carolina, Chapel Hill, NC USA; 2 Van Andel Institute, Grand Rapids, MI USA; 3 Nationwide Children’s Hospital, Columbus, OH USA; 4 Dana-Farber Cancer Institute, Harvard Medical School, Boston, MA USA; 5 Division of Breast Surgery, Brigham and Women’s Hospital, Boston, MA USA; 6 SOLTI Cancer Research Group, Barcelona, Spain; 7 Vanderbilt University Medical Center, Nashville, TN USA; 8 Medical University of South Carolina, Charleston, SC USA; 9 Boehringer Ingelheim, Ridgefield, CT USA; 10 Johns Hopkins University, Baltimore, MD USA; 11 Baylor College of Medicine, Houston, TX USA; 12 Translational Breast Cancer Research Consortium, Baltimore, USA; 13 Duke University, Durham, NC USA; 14 University of Chicago, Chicago, IL USA; 15 Georgetown University, Washington, DC USA; 16 Indiana University School of Medicine, Indianapolis, IN USA; 17 Mayo Clinic, Rochester, MN USA; 18 UPMC Hillman Cancer Center, University of Pittsburgh, Pittsburgh, PA USA; 19 Pittsburgh Supercomputing Center, Carnegie Mellon University, Pittsburgh, PA USA; 20 Memorial Sloan Kettering Cancer Center, New York, NY USA; 21 MD Anderson Cancer Center, Houston, TX USA; 22 Fred Hutchinson Cancer Research Center, University of Washington, Seattle, WA USA

**Keywords:** Cancer epigenetics, Cancer genomics, Breast cancer, Cancer

## Abstract

The AURORA US Metastasis Project was established with the goal to identify molecular features associated with metastasis. We assayed 55 females with metastatic breast cancer (51 primary cancers and 102 metastases) by RNA sequencing, tumor/germline DNA exome and low-pass whole-genome sequencing and global DNA methylation microarrays. Expression subtype changes were observed in ~30% of samples and were coincident with DNA clonality shifts, especially involving HER2. Downregulation of estrogen receptor (ER)-mediated cell–cell adhesion genes through DNA methylation mechanisms was observed in metastases. Microenvironment differences varied according to tumor subtype; the ER^+^/luminal subtype had lower fibroblast and endothelial content, while triple-negative breast cancer/basal metastases showed a decrease in B and T cells. In 17% of metastases, DNA hypermethylation and/or focal deletions were identified near *HLA-A* and were associated with reduced expression and lower immune cell infiltrates, especially in brain and liver metastases. These findings could have implications for treating individuals with metastatic breast cancer with immune- and HER2-targeting therapies.

## Abstract (web summary)

Perou and colleagues perform genomic, transcriptomic and epigenetic analyses on pairs of primary and metastatic breast tumors, detecting subtype switching and changes in immune signatures and DNA methylation patterns associated with metastasis.

## Main

A great deal of effort has gone into understanding the molecular causes of metastatic breast cancer (MBC), to which ~45,000 individuals per year succumb in the United States^1^. An early focus on metastatic disease has been to identify somatic DNA-based alterations that might be unique to this setting and/or that may be clinically actionable, especially when metastasis surgical resection may not be a viable option. Numerous seminal publications on MBC genomics have shown that almost no recurrent mutations are unique to the metastatic landscape, with perhaps the exception of *ESR1* mutations, most of which are thought to be tied to endocrine therapy resistance^2–5^. Instead, modestly increased frequencies of known pathogenic somatic variants (that is, *TP53*, *PTEN* and *RB1*) and/or altered mutational signatures have been identified in metastases^6^, as have similarly modest increases in the frequency of DNA amplifications/deletions^2,7^. Thus, much of the aggressive behavior of metastatic disease remains unexplained by DNA-based changes, invoking the need for a multiomic evaluation of this disease setting. Among the most impactful therapeutic advances in MBC has been the development and use of CDK4/CDK6 inhibitors^8–10^, novel HER2-directed agents^11,12^ and immune checkpoint inhibitors (ICIs) targeting CTLA4, PD-1 or PD-L1 (refs. 13–15). These latter therapies target the immunosuppressive tumor immune microenvironment, thus highlighting the importance of non-tumor-intrinsic factors as a major determinant of disease outcomes. Human leukocyte antigen (HLA) class I downregulation could also be a barrier to effective T cell-based immunotherapy. Alterations in major histocompatibility complex (MHC) class I molecules can prevent tumor cells from being recognized by cytotoxic lymphocytes^16–18^. In BC, ICIs have gained a role in both the early-stage and metastatic settings, albeit with some mixed results^19^, thus highlighting the need for an improved understanding of the MBC-intrinsic and MBC-extrinsic landscapes. Here, we present results from the AURORA US retrospective metastatic project that, along with the AURORA EU project^3^, represent two of the most ambitious programs to improve our molecular knowledge of MBCs.

## Results

### Clinical features of the cohort and global genomic patterns

**Fig. 1:**
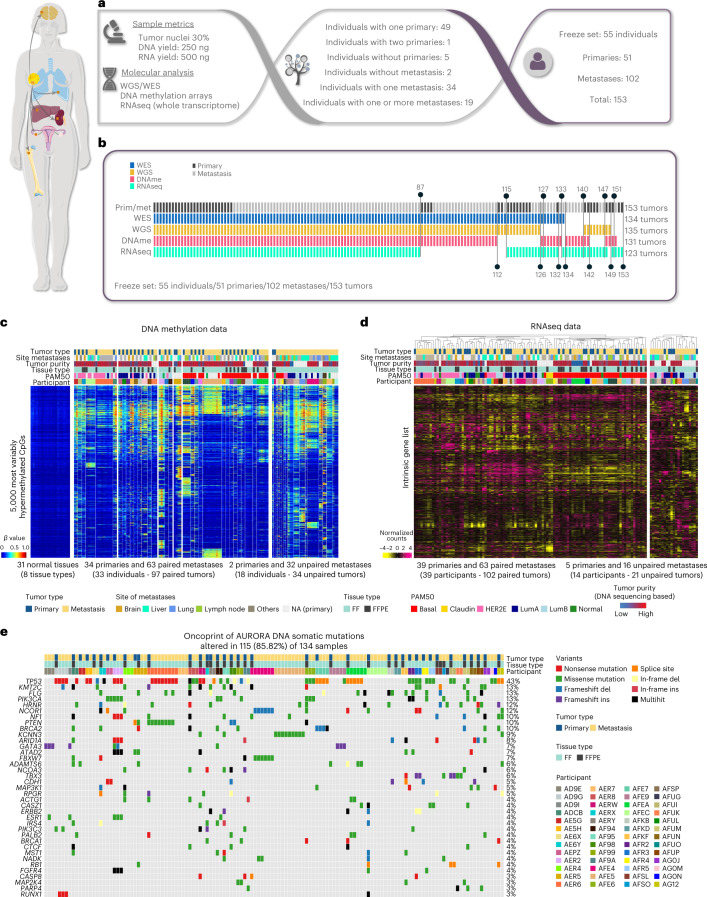
Study design and global genomic patterns of metastatic breast tumors. **a**, Cohort description of the AURORA Metastatic Project. **b**, Diagram of the shared or individual tumor DNA methylation, WGS/whole-exome sequencing (WES) and RNAseq data successfully performed on each of the 55 participants; DNAme, DNA methylation; prim, primary; met, metastasis. **c**, Global profiling of the DNA methylation landscape using the top 5,000 most variable cancer-associated hypermethylated CpGs in 97 paired and 34 unpaired primary and metastatic tumors. Samples were intentionally ordered by participant to visually inspect the within-participant conservation of DNA methylation patterns. **d**, Supervised hierarchical cluster analysis of 102 paired and 21 unpaired primary and metastatic RNA-sequenced tumors using the so-called 1,900 intrinsic gene list (~1710 genes found in this dataset)^21^. **e**, OncoPrint panel of DNA somatic mutations displaying 37 of the most frequently mutated genes in 41 primary and 93 metastatic tumors. The percentage on the right indicates the mutation frequency of each gene across samples; LumA, Luminal A; LumB, Luminal B, Claudin, Claudin-low; normal, normal-like; Del, deletion; Ins, insertion. This figure was partly generated using Servier Medical Art, provided by Servier, licensed under a Creative Commons Attribution 3.0 unported license (smart.servier.com).

**Extended Data Fig. 1:**
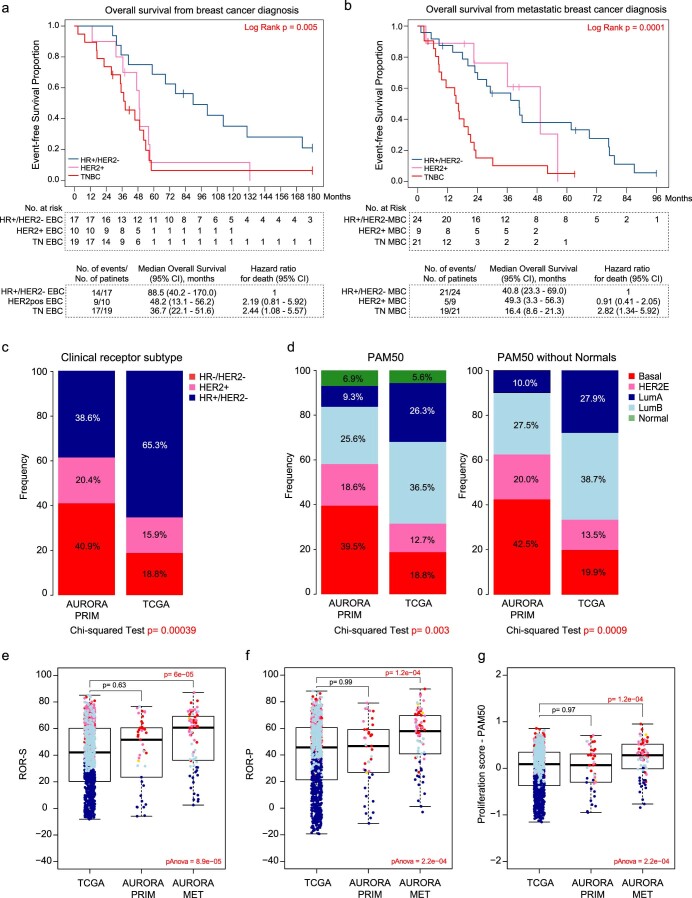
Survival outcomes according to clinical subtypes of AURORA cohort. **a**. Kaplan-Meier, log-rank test and Cox proportional hazards regression model methods were used to study the overall survival from breast cancer diagnosis (‘First Primary Receptor at diagnosis’ column of Supplementary Table 2) in HER2 positive (HER2+, n = 10 patients), Hormone receptor positive and HER2 negative (HR + /HER2-, n = 17 patients) and TNBC (triple-negative breast cancers, n = 19 patients). **b**. Kaplan-Meier, the log-rank test and Cox proportional hazards regression model to study the overall survival from metastatic breast cancer diagnosis (‘Metastasis original receptors’ column of Supplementary Table 2) in HER2 + (n = 9 patients), HR + /HER2- (n = 24 patients) and TNBC (n = 21 patients). In absence of HR/HER2 status in the metastatic relapse we used the data from the most recent biopsy. **c-d**. Frequency bar chart displaying the frequency of clinical subtype (**c**) and molecular subtype (**d**) in AURORA (n = 123 tumors) compared with TCGA (n = 1027 tumors). In the AURORA cohort, we assigned ER and HER2 clinical status to those samples that had missing clinical values using the mRNA surrogates. **e-g**. Boxplot displaying the risk of recurrence based on subtype (ROR-S) (**e**) and proliferation (ROR-P) (**f**) and Proliferation score from PAM50 predictor (**g**) comparing TCGA primary tumors (n = 1027 tumors) vs AURORA primary tumors (n = 44 tumors) vs AURORA metastatic tumors (n = 70 tumors). Statistically significant values are highlighted in red. Comparison between more than 2 groups was performed by ANOVA with post hoc Tukey’s test, one-sided (panels e, f, and g). Normal-like samples were removed from this analysis. Box-and-whisker plots from panels e, f, and g, display the median value on each bar, showing the lower and upper quartile range of the data (Q1 to Q3) and data outliers. The whiskers represent the lines from the minimum value to Q1 and Q3 to the maximum value. EBC, early breast cancer; MBC, metastatic breast cancer; confidence interval (CI). Statistically significant values are highlighted in red.

**Extended Data Fig. 2:**
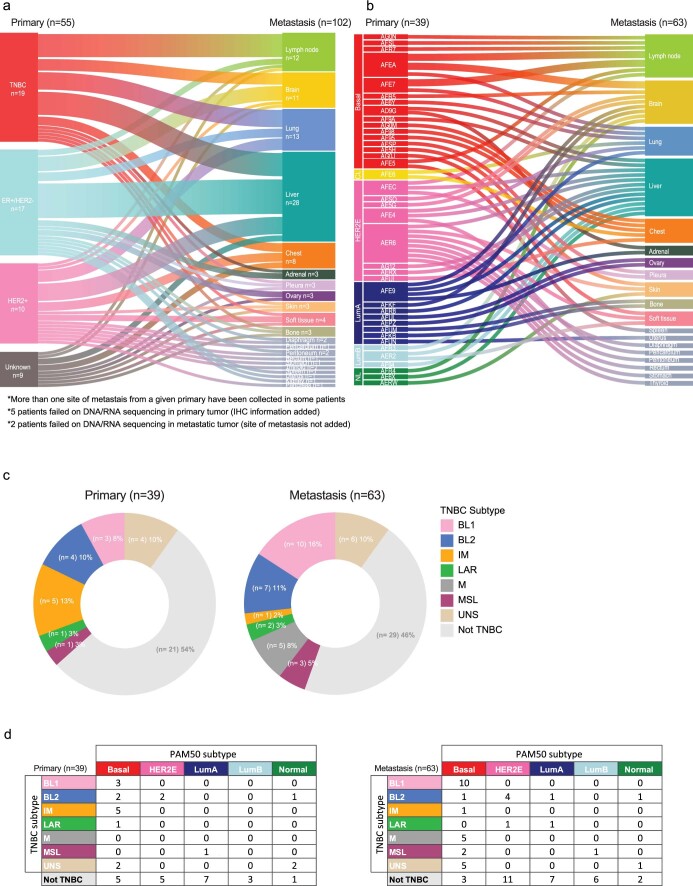
Clinical subtype and molecular subtype distribution according to site of metastasis. **a**. Distribution of the 55 diagnosed primary tumors (n = 39 primaries) by clinical receptor status (TNBC, ER+/HER2-, HER2+, and unknown, left side) linked to their anatomic sites of metastasis (n = 63 metastases). Clinical receptor status at the time of first primary diagnosis (‘First Primary Receptors’ column of Supplementary Table 2). **b**. Distribution of 39 diagnosed primary tumors by gene expression-based intrinsic molecular subtype when available (left) linked to their anatomic sites of metastasis (right). **c**. TNBC and non-TNBC subtype proportions of primary (left, n = 39 primaries) and paired metastatic (right, n = 64 metastases) tumors by TNBCtype^25^. **d**. Comparison of subtype classifications between TNBC subtype and PAM50 of primary (left, 39 primaries) and paired metastatic (right, 63 metastases) tumors. LumA, Luminal A; LumB, Luminal B; CL, Claudin-low; NL, normal-like; BL1, basal-like 1; BL2, basal-like 2; IM, immunomodulatory; LAR, luminal androgen receptor; M, mesenchymal-like; MSL, mesenchymal stem-like.

A consortium of academic medical centers in the United States was formed (AURORA US Metastatic Project) based on the infrastructure of the Translational Breast Cancer Research Consortium to pursue a multiplatform genomic study of matched metastatic and primary BCs, similar to The Cancer Genome Atlas (TCGA) effort on primary BCs^20^. Eligibility criteria for this retrospective study included the availability of a fresh-frozen (FF) metastatic specimen, its associated primary tumor (FF or formalin-fixed paraffin-embedded (FFPE) samples), a source of normal DNA and corresponding tumor pathology and molecular analyte metrics (Fig. 1a). These requirements identified 55 individuals, including 19 individuals with more than one metastasis analyzed; 20 participant samples were collected at autopsy (representing the individuals with more than one metastasis). The clinical demographics of this group constituted a young cohort with a median age at primary diagnosis of 49 years, of which 18% were African American and 7% were of Hispanic ethnicity. In the metastatic setting, these individuals received a median of three lines of systemic therapy. As might be expected, the overall survival of these individuals was generally poor and differed according to clinical subtype (Extended Data Fig. 1a,b). The median overall survival from BC diagnosis was 4.5 years and from metastatic diagnosis was ~2 years. Compared to TCGA primary tumors, the AURORA cohort also had a higher frequency of triple-negative BC (TNBC) and basal-like primary tumors (Extended Data Fig. 1c,d). The risk of recurrence score-based genomic features and the proliferation score itself were higher in metastatic samples than in AURORA and TCGA primary tumors (Extended Data Fig. 1e–g). Metastases were obtained from multiple sites, with the most common being liver (*n* = 28), lung (*n* = 13), lymph nodes (*n* = 12), brain (*n* = 11) and 16 other sites; the relationships between clinical or genomic subtype and site of metastasis are shown in Extended Data Fig. 2. Additional clinical demographics are shown in Supplementary Table 1.

**Extended Data Fig. 3:**
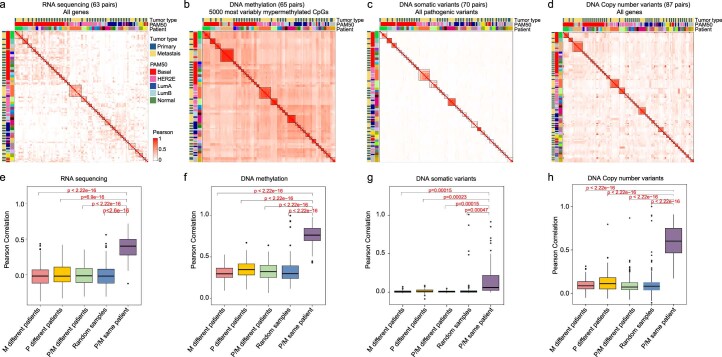
Correlation analysis between paired data in each genomic approach. **a-d**. Correlation heatmap representing the correlation matrix of (**a)** RNAseq data, n = 63 tumor pairs (gene expression values) (**b**) DNA methylation data, n = 65 tumor pairs (ꞵ-values), (**c**) DNA somatic variants, n = 20 tumor pairs (binary data: 1, mutated and 0, non-mutated) and (**d**) DNA copy number variants, n = 87 tumor pairs (gene-specific denoised log2 copy-ratios) of paired primary and metastatic tumors. The relationship between variables has been calculated using the Pearson correlation coefficient. **e-h**. Comparison of Pearson correlation means between primary and paired metastasis, random primary and metastatic tumors, primaries and metastasis belonging to different patients, primary samples belonging to different patients, and metastasis samples belonging to different patients of (**e**) RNAseq data, n = 63 tumor pairs (gene expression values) (**f**) DNA methylation data, n = 65 tumor pairs (ꞵ-values), (**g**) DNA somatic variants, n = 20 tumor pairs (binary data: 1, mutated, 0 non-mutated) and (**h)** DNA copy number variants, n = 87 tumor pairs (gene-specific denoised log2 copy-ratios). P values between groups were calculated using *t-*test, two-sided (panels, e, f, g, and h). In panels e, f, g, and h, all Box-and-whisker plots display the median value on each bar, showing the lower and upper quartile range of the data (Q1 to Q3) and data outliers. The whiskers represent the lines from the minimum value to Q1 and Q3 to the maximum value. P, Primary; M, metastasis.

Tumor DNA and RNA were isolated from each specimen and used in four different assays: DNA exome and low-pass whole-genome sequencing (WGS; tumor and normal), whole-transcriptome RNA sequencing (RNAseq) using rRNA depletion and DNA methylation microarrays. In total, 88 of 153 specimens had all four assays successfully performed, and 141 of 153 had three of four completed (Fig. 1b); this multiplatform genomic dataset of 102 metastases and 51 paired primary tumors thus represents an unprecedented resource for the study of MBC. Global profiling of the DNA methylation landscape using the top 5,000 most variably methylated CpGs displaying cancer-associated hypermethylation showed a remarkable conservation of overall methylation profiles within most primary tumor–metastasis pairs (Fig. 1c); indeed, 32 of 36 tumor–metastasis pairs showed the highest correlation to each other. Similar to the DNA methylation findings, gene expression-based hierarchical clustering using a 1,710-gene breast tumor ‘intrinsic’ list^21^ also identified the individuality of each primary tumor–metastasis pair, where 31 of 39 pairs were coclustered in the dendrogram (Fig. 1d), as seen in other studies of metastases^22,23^. To quantify the degree of similarity between pairs, we compared the average correlation between random pairs and matched pairs (Extended Data Fig. 3a–d). These comparisons revealed that overall, primary tumors are more similar to paired metastatic samples than to other breast tumors. Lastly, the somatic mutation landscape identified *TP53*, *KMT2C*, *FLG* and *PIK3CA* as the most frequently mutated genes, together with the presence of *ESR1* mutations in metastases from four individuals with estrogen receptor-positive (ER^+^) BC AF94, AER2, AD9I and AD9E (Fig. 1e). Similarly, most somatic mutations within bona fide BC driver genes (defined in TCGA) found in AURORA primary tumors were also present in the paired metastasis (Fig. 1e). *TP53* and *FLG* genes were more frequently mutated in metastases than in primary tumors (66% versus 33% (*P* = 0.006) and 28% versus 3% (*P* = 0.003), respectively); however, this finding did not reach statistical significance after false discovery rate (FDR) adjustment. For the somatic DNA copy number landscape, we calculated 533 recurrent DNA segment-level scores (Methods and Supplementary Table 8) and observed that 11 segments were found to be more frequently amplified in metastases (*q* < 0.05). Of these 11 segments, all overlapped an amplified region found in Bertucci et al.^2^, and 2 overlapped amplified regions found in Aftimos et al.^3^ related to *MYC* and *MDM4* amplifications.

### Gene expression subtype switching and genomic signature differences

**Fig. 2:**
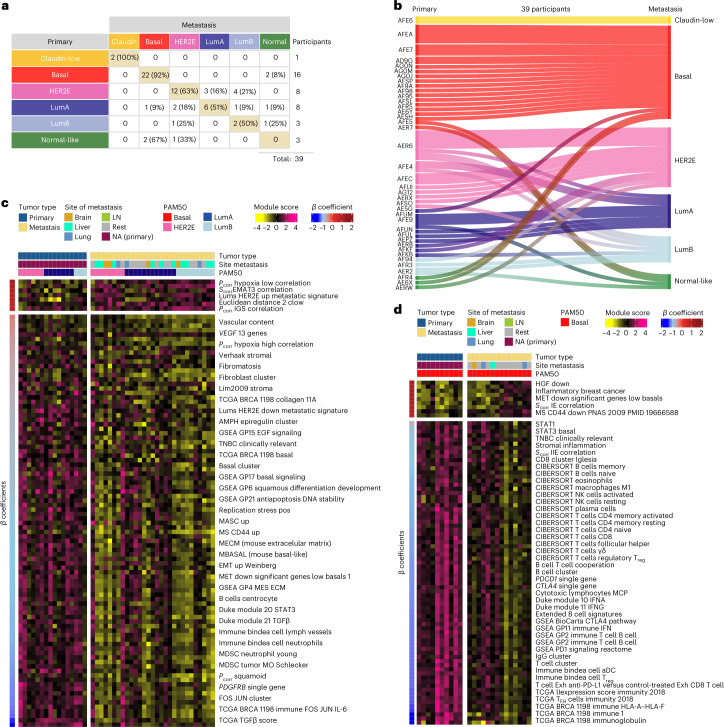
Subtype switching and supervised analysis of gene expression signatures between primary and metastatic tumors. **a**, Overall molecular intrinsic subtype change between 39 participant-matched primary breast and 1 or more metastatic tumors. **b**, Participant-specific molecular subtype changes in 39 participant-matched primary breast and 1 or more metastatic tumors. **c**,**d**, Heat maps of some representative signatures that are significantly different between primary and metastatic tumors in luminal/HER2E (*n* = 16 primary versus 29 metastatic tumors; **c**) and basal-like only subtypes (*n* = 10 primary versus 14 metastatic tumors; **d**). Significance of the differences between primary tumors and metastases were calculated using LMMs (*q* < 0.01). Significant signatures are row ordered from high to low according to *ꞵ*-coefficients (or regression coefficients) and divided according to upregulated (positive) or downregulated (negative) in metastasis. Individuals are column ordered according to PAM50 molecular subtype and divided according to primary tumor and metastasis. Signature scores were calculated in the level 4 RNAseq data (Methods). Normal-like tumors and post-treatment primaries were removed from the analysis in the AURORA cohort. For more information about the background/origin of the signatures listed in **c** and **d**, see Supplementary Table 3, sheet 2. LumA, Luminal A; LumB, Luminal B; LN, lymph node.

To evaluate gene expression differences between primary tumors and their metastases, we performed PAM50 molecular subtyping from RNAseq data for each of the 123 specimens^21,24^ and tested subtype concordance within each individual (Fig. 2a,b). Of the 39 RNAseq cases tested, 13 of 39 showed subtype ‘switching’ between a primary tumor and its metastasis. We note that the normal-like distinction typically reflects low tumor cellularity (tumor cellularity and ESTIMATES scores are in Supplementary Table 2); therefore, if we disregard switching to or from the normal-like group, then the basal-like phenotype is the most stable, with 15 of 16 pairs being basal-like in all specimens. Conversely, the ‘luminal’ phenotypes that include Luminal A (LumA), Luminal B (LumB) and HER2 enriched (HER2E), experienced subtype switching in 8 of 19 individuals. We also performed TNBC subtyping^25^ on the TNBC samples (Extended Data Fig. 2), and, interestingly, we observed a decreased frequency of the immunomodulatory (IM) subtype, from 13% in the primary tumor to 2% in the metastatic setting (Supplementary Table 2).

**Extended Data Fig. 4:**
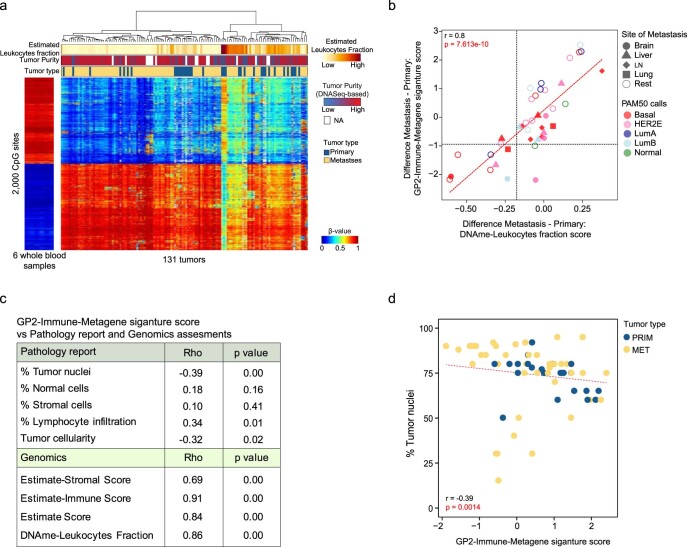
Correlation between tumor cellularity metrics and immune signatures. **a**. Supervised hierarchical clustering of the top 1,000 leukocyte-specifically methylated probes and the bottom 1,000 tumor tissue-specifically methylated probes, after ranking all probes based on the mean leukocytes - mean tumor tissues. For the 133 tumors, association is shown with tumor type, tumor purity, and estimated leukocyte fraction scores^26,27^. **b**. Pearson correlation between the difference (Metastasis – Primary gene expression values, n = 40 tumor pairs) of the Leukocyte fraction scores and GP2-Immune-Metagene signature scores (calculated from the Level 4 RNAseq data). Higher scores mean higher expression in metastasis compared to primary tumors. Correlation was measured using the Pearson correlation coefficient (r) and p values were used to assess the significance of the correlation. **c**. Spearman correlations (Rho) between GP2-Immune-Metagene signature scores and several pathology-determined scores (% Tumor nuclei, % of normal cells, % of Stromal cells, % Lymphocyte infiltration and tumor cellularity) or genomic scores (Estimate-Stromal scores, Estimate-Immune Score and Estimate Score using ESTIMATE method^57^ using 65 tumors (23 primary and 42 metastasis). Rho (spearman correlation coefficient, ρ), p values were used to assess the significance of the correlation. **d**. Pearson correlation (r) of GP2-Immune-Metagene signature score and % of Tumor nuclei from pathology report using 65 tumors (23 primary and 42 metastasis). P values were used to assess the significance of the correlation. Statistically significant values are highlighted in red. GP2-Immune-Metagene signature scores were calculated from the Level 4 RNAseq data (see Methods). LumA, Luminal A; LumB, Luminal B.

**Extended Data Fig. 5:**
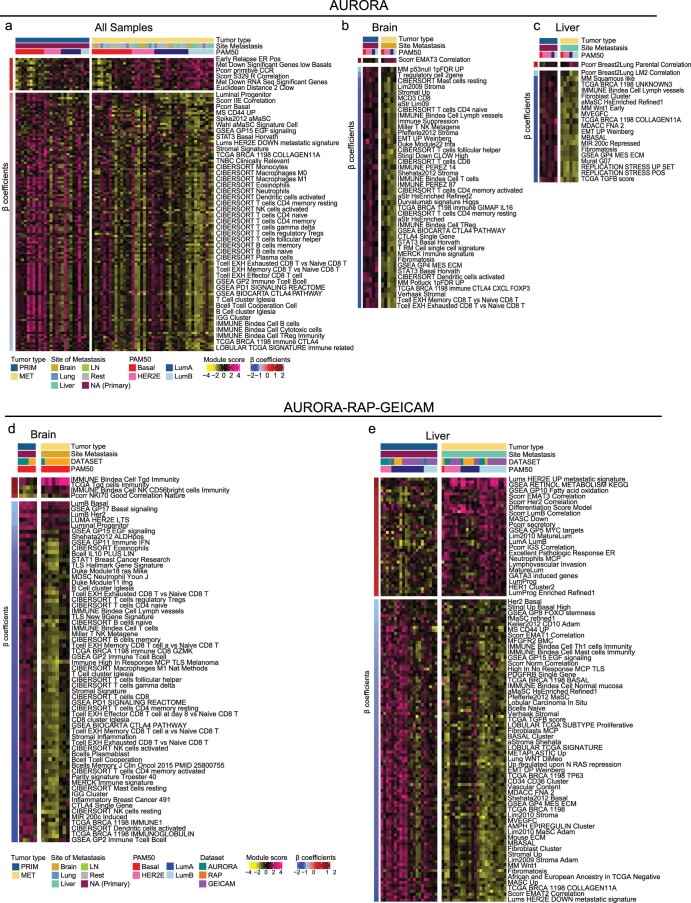
Supervised analysis of gene expression signatures according to site of metastasis in AURORA or combined AURORA-RAP-GEICAM cohorts. a. Heatmap depicting the differentially expressed (DE) signatures between primary (n = 26) and metastasis (n = 69) in the AURORA cohort using all samples. b. Heatmap depicting the DE signatures between paired primary (n = 5) and brain metastasis (n = 5) in the AURORA cohort. c. Heatmap depicting the DE signatures between paired primary (n = 6) and liver metastasis (n = 6) in the AURORA cohort. d. Heatmap depicting the DE signatures between basal-like paired primary (n = 5) and brain metastasis (n = 8) in the AURORA-RAP-GEICAM cohort. d. Heatmap depicting the DE signatures between luminals (LumA, LumB, and HER2E) paired primary (n = 21) and liver metastasis (n = 24) in the AURORA-RAP-GEICAM cohort. Significance of the differences between primary and metastasis was calculated using linear mixed models (q < 0.05 in AURORA and q < 0.02 in AURORA-RAP-GEICAM). Significant signatures are row ordered from high to low according to β coefficients (or regression coefficients) and divided according to upregulated (positive) or downregulated (negative) in metastasis. Patients are column ordered according to PAM50 molecular subtype and divided according to primary and metastasis. Signatures scores were calculated in the Level 4 RNAseq data (see Methods). Normal-like tumors and post-treatment primaries were removed from the analysis. For more information about the background/origin of the signatures listed in this figure, see Supplementary Table 3, sheet 2. LumA, Luminal A; LumB, Luminal B; LN, lymph node.

We next performed additional RNAseq-based statistical analyses specifically comparing primary tumors to various groupings of the metastases. We first transformed the gene expression data into a set of 749 previously published gene expression signatures representing many features of tumor cells and their microenvironment, including >100 signatures of immune cells, which showed significant correlation with pathologist-assessed percent immune cell infiltration and with DNA methylation-based assessments of leukocyte infiltration^26,27^ (Extended Data Fig. 4a–d); the complete list of signatures is shown in Supplementary Table 2. Throughout our analyses, we relied on multiple validated immune cell signatures, including many that have shown prognostic and predictive value^24,27–29^, as our main measures of immune cell presence/involvement. These immune signatures include many focused on adaptive immunity and include CIBERSORT signatures of T cells and B cells^30^ and signatures of cooperating immune cells, including an IgG signature^31^, a B cell/T cell cooperativity signature^32^ and a GP2-immune-metagene signature (Methods). We performed supervised analyses of all primary tumors versus all metastases using this library of signatures and identified 135 signatures as being differentially expressed (*q* < 0.05; Extended Data Fig. 5a), including signatures of fibroblasts/stromal cells and endothelial cells and many adaptive immunity signatures as being lower in metastases. However, when supervised analyses were performed within a gene expression subtype, which is known to associate with the likelihood of metastasis^33,34^, then subtype-specific differences were observed (Fig. 2c,d). Specifically, luminal/ER^+^ subtype metastases (LumA, LumB and HER2E combined) showed low expression of fibroblast and endothelial signatures, and very few adaptive immune features were different. Conversely, basal-like/TNBC metastases had significantly lower expression of adaptive immune features, including multiple T cell-, B cell-, natural killer (NK) cell- and HLA-related signatures, while signatures of fibroblasts and endothelial cells were unchanged (Fig. 2d).

We next asked if there were expression signature differences according to site of metastasis, and here we focused on the three most frequent sites (that is, liver, lung and brain). Using only the AURORA dataset, testing of primary versus paired brain metastases yielded 48 signatures as being lower in brain metastases, most of which were features of immunity and fibroblasts/stromal cells (Extended Data Fig. 5b). Supervised analysis of liver metastases versus their primary tumors yielded 22 signatures as differentially expressed (Extended Data Fig. 5c), while a similar analysis of lung metastases yielded no significant signatures. The small number of differentially expressed features suggested that we may be limited by our sample size; therefore, we obtained a second dataset of primary tumor–metastasis pairs from our University of North Carolina (UNC) Rapid Autopsy Program (RAP; 2 primary tumor–metastasis pairs, 10 primary tumor–multiple metastasis pairs and 22 unpaired metastases represented by 82 specimens) and a third dataset from the public domain that had 102 primary tumor–metastasis pairs from the GEICAM/2009-03 ConvertHER (GEICAM) trial^22^. Using this RNAseq combined cohort to compare primary tumors and liver metastases (*n* = 58 tumors, 27 primary tumors and 31 metastases) yielded a larger set of significant signatures that included many adaptive immunity signatures as being lower in liver metastasis (Extended Data Fig. 5e). In addition, the combined cohort allowed us to refine our analysis of brain metastases in the setting of the basal-like/TNBC phenotype (*n* = 13 tumors, 5 primary tumors and 8 metastases), which also yielded more significant signatures, including upregulated cell differentiation-related signatures and lower immune and stromal-related signatures (Extended Data Fig. 5d). Lastly, the combined analysis of primary lung metastases (*n* = 36 tumors, 18 primary tumors and 17 metastases) still yielded no significant signatures.

**Fig. 3:**
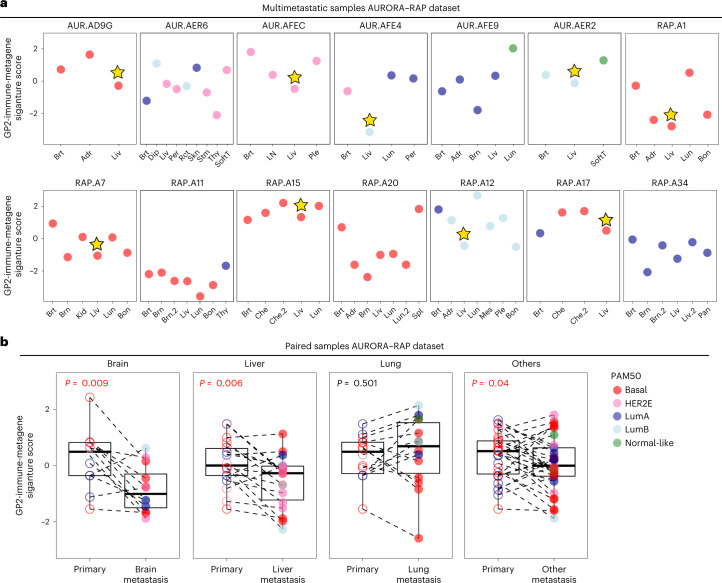
Individuals with multiple metastases were examined for immune features in the AURORA–RAP combined cohort. **a**, Gene expression signature scores of GP2-immune-metagene are shown according to individual specimens from participants with at least two metastases analyzed by RNAseq data (*n* = 14 individuals). The star indicates liver specimens with the lowest expression of signature. **b**, Expression changes between paired primary tumors and liver (36 pairs), brain (15 pairs), lung (21 pairs) or ‘rest’ (110 pairs) metastases of the GP2-immune-metagene signature (individuals with more than one metastasis in the same organ were averaged). Comparisons between two paired groups were performed by a two-sided paired samples Wilcoxon test. Statistically significant values are highlighted in red. All box and whisker plots display the median value on each bar, showing the lower and upper quartile range of the data (Q1 to Q3). The whiskers represent the lines from the minimum value to Q1 and Q3 to the maximum value; LumA, Luminal A, ; LumB, Luminal B; Brt, breast; Adr, adrenal; Liv, liver; Dip, diaphragm; Per, peritoneum; Rct, rectum, Skn, skin; Stm, stomach; Thy, thyroid; SoftT, soft tissue; LN, lymph node; Ple, pleura; Lun, lung; Brn, brain; Bon, bone; Kid, kidney; Che, chest; Spl, spleen; Mes, mesentery; Pan, pancreas; AUR, AURORA.

These comparative analyses suggest that immune features may systematically vary according to site of metastasis. To directly address this hypothesis, we took advantage of the combined AURORA–RAP datasets that contain 14 participants with at least two metastases analyzed by RNAseq (one of which is from the liver) to examine immune signature levels in different metastatic sites within the same individual. This analysis showed that in 9 of 14 individuals, the lowest levels of the GP2-immune-metagene signature were in liver metastases (Fig. 3a,b), and in many of these individuals, this immune signature is lower in the liver metastases than in the matched primary tumor but is often higher in lung metastases (Fig. 3a,b). Next, we performed statistical testing using the combined AURORA–RAP–GEICAM cohort and comparing liver to lung metastases and liver to lymph node metastases, both of which demonstrated significantly decreased immune signatures in liver metastases (Supplementary Table 3). We also compared liver metastases and brain metastases and saw 76 differential signatures that were primarily non-immune related (except for higher γδ T cells in brain metastases). When brain metastases were compared to lung or lymph node metastases, brain metastases also demonstrated lower expression of immune-associated signatures.

Finally, to evaluate the gene expression signatures as a predictive variable in survival analysis, we performed Cox proportional hazard models from time of BC diagnosis to death (overall survival) in the AURORA cohort. The major determinant of survival in this cohort was, as might be expected, a luminal/ER^+^-related (better outcomes) signature versus a basal-like related phenotype signature (worse outcomes). When adjusting for clinical or molecular subtype, the main survival findings were immune-related signatures that predicted better outcomes (Supplementary Table 4).

### *HLA-A* dysregulation and impact on antitumor immunity

**Fig. 4:**
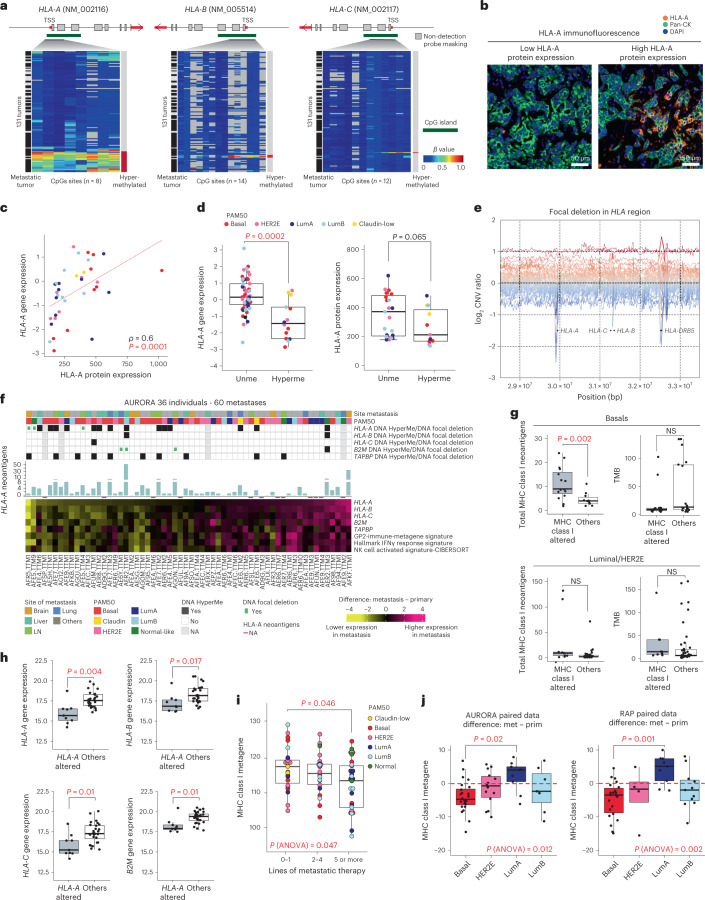
*HLA-A* dysregulation and impact on immune-related features in metastatic tumors. **a**, Hypermethylated CpG sites in *HLA-A* (8 CpG sites), *HLA-B* (14 CpG sites) and *HLA-C* (12 CpG sites) of 133 primary and metastatic tumors; TSS, transcription start site. **b**, Representative images of 37 metastatic samples showing HLA-A immunofluorescence staining for two different levels of HLA-A protein expression (top third and bottom third). HLA-A protein expression values were divided into tertiles on the basis of low (lower third), intermediate (middle third) or high intensity (upper third). **c**, Correlation analysis of HLA-A protein expression and *HLA-A* gene expression values (*n* = 37 metastases). The correlation was measured using the Spearman correlation coefficient. **d**, Box plots of *HLA-A* mRNA gene expression levels in metastases (left; *n* = 75 metastatic tumors) and HLA-A protein expression (right; *n* = 34 metastatic tumors) according to DNA methylation status when data were available. **e**, *HLA-A*, *HLA-B*, *HLA-C* and *HLA-DRB5* focal deletions in the *HLA* region of 49 individuals. **f**, Heat map representation of the difference in *HLA-A*, *HLA-B*, *HLA-C*, *B2M* and *TAPBP* gene expression values and GP2-immune-metagene and hallmark interferon-γ (IFNγ) response gene signature scores, calculated between paired primary (*n* = 36) and metastatic (*n* = 60) tumors. Normal-like paired and unpaired tumors were removed from this analysis (paired normal and unpaired group from the ‘Pairs-PAM50-Prim’ column of Supplementary Table 2). Gene and signature scores are ordered according to *HLA-A* gene expression changes. For the 60 metastases, the association is shown with *HLA-A*, *HLA-B*, *HLA-C*, *B2M* and *TAPBP* gene methylation/DNA focal deletion status, PAM50 and site of metastasis; NK, natural killer. **g**, Left, MHC class I-associated neoantigen levels in MHC class I-altered tumors (*HLA-A*, *HLA-B*, *HLA-C*, *B2M* and *TAPBP* hypermethylation or focal deletion) versus non-altered tumors (Others) when data were available (basal-like tumors: *n* = 25, 5 primaries and 20 metastases; luminal/HER2E tumors: *n* = 39, 9 primaries and 30 metastases). Right, TMB in MHC class I-altered tumors versus in other tumors when data were available (basal-like tumors: *n* = 35, 11 primaries and 24 metastases; luminal/HER2E tumors: *n* = 52, 15 primaries and 37 metastases); NS, not significant. **h**, *HLA-A*, *HLA-B*, *HLA-C* and *B2M* gene expression values are shown in *HLA-A*-altered versus other tumors when data were available (*n* = 37, 13 primaries and 24 metastases). **i**, MHC class I metagene signature scores according to lines of therapies in metastatic samples (*N* = 77). **j**, MHC class I metagene signature score differences between primary and metastatic tumors according to molecular subtype in AURORA (*n* = 46) and RAP (*n* = 57) cohorts. Normal-like tumors were removed from the analysis. All box and whisker plots of the figure display the median value on each bar, showing the lower and upper quartile range of the data (Q1 to Q3) and data outliers. The whiskers represent the lines from the minimum value to Q1 and Q3 to the maximum value. All comparisons between more than two groups were performed by ANOVA with a post hoc Tukey test (one sided), and *P* values are shown in red (**i** and **j**). Comparison between only two groups was performed by unpaired Mann–Whitney test (two sided), and significant *P* values are highlighted in red (**d**, **g** and **h**). LumA, Luminal A; LumB, Luminal B; LN, lymph node; Unme, unmethylated; HyperMe, hypermethylated.

**Extended Data Fig. 6:**
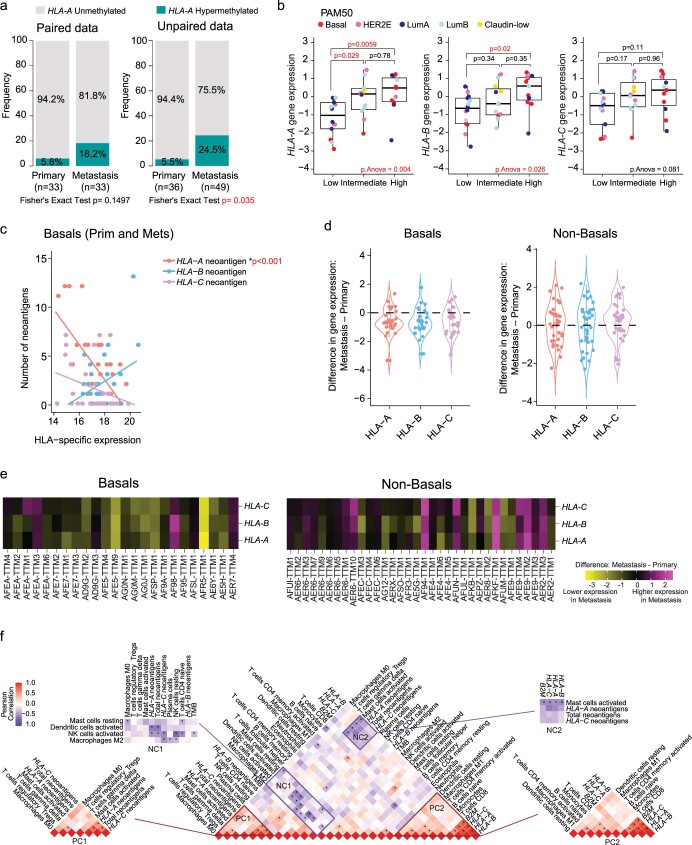
HLA-A gene and protein expression levels in metastatic samples and impact on immune-related features in metastatic tumors. **a**. Bar plot depicting the frequency of HLA-A Unmethylated, and HLA-A methylated samples divided by primary and metastatic tumors. Fisher’s exact test was used to compare the proportion of categories (the number of samples is shown in the figure). **b**. Boxplots of *HLA-A, -B*, and *C* mRNA gene expression levels and according to HLA-A protein expression (n = 37 metastasis). HLA-A protein expression values were divided into tertiles on the basis of low (lower third; n = 14), intermediate (middle third; n = 12), or high intensity (upper third, n = 11). Comparison between more than 2 groups was performed by ANOVA with post hoc Tukey’s test, one-sided. Statistically significant values are highlighted in red. Comparisons between 2 paired groups were performed by *t*-test. Comparison between more than 2 groups was performed by ANOVA with post hoc Tukey’s test, one-sided. All Box-and-whisker plots display the median value on each bar, showing the lower and upper quartile range of the data (Q1 to Q3) and data outliers. The whiskers represent the lines from the minimum value to Q1 and Q3 to the maximum value. Normal-like samples were removed from this analysis. Statistically significant values are highlighted in red. **c**. Linear relationship between number of neoantigens and *HLA-A*, -*B* and *C* gene expression Level 4 RNAseq data (see Methods) of basal-like only primary and metastatic tumors. The correlation was measured using the Pearson correlation coefficient. **d**. Violin plots showing changes in gene expression for *HLA-A*, -*B*, and -*C* between primary and metastatic samples (Difference: Metastasis – Primary gene expression values) in basals (right panel, n = 34 tumors) and luminals/HER2E metastatic tumors (right panel, n = 34 tumors). **e**. Patient-specific changes in gene expression for *HLA-A*, -*B*, and -*C* between primary and metastatic samples (Difference: Metastasis – Primary gene expression values) in basal-likes, (left panel, n = 24 tumors) and luminals/HER2E metastatic tumors (right panel, n = 34 tumors) of AURORA cohort. Normal-like paired and unpaired tumors were removed from this analysis (Paired Normal and unpaired group from the ‘Pairs-PAM50-Prim’ column of Supplementary Table 2). **f**. Correlation matrix and unsupervised hierarchical clustering of CIBERSORTx-based immune-cell scores in basal-like samples (n = 42, 17 primary and 25 Metastasis). Positive clusters (PC1 and PC2) and negative clusters (NC1 and NC2) reflect the highest or lowest correlated immune-related signature scores per CIBERSORTx. Correlation was measured using the Pearson correlation coefficient and p values <0.05 are shown as (*). ns, non-significant. Prim, primary; Met, metastasis; LumA, Luminal A; LumB, Luminal B.

The decreased expression of an *HLA* metagene signature in basal-like/TNBC metastases led us to examine the multiplatform data of the individual genes comprising this signature, including *HLA-A*, *HLA-B*, *HLA-C* and *B2M*. Examining promoter CpG islands for *HLA-A*, *HLA-B*, *HLA-C* and *B2M*, we identified *HLA-A* methylation in 23 tumors (12 individuals), and *HLA-A* promoter methylation was significantly more frequent in metastases than in primary tumors when comparing unpaired data (*P* = 0.035), whereas paired analyses were not significant but trending in the same direction (Fig. 4a and Extended Data Fig. 6a). By contrast, only three tumors (one individual) demonstrated methylation at *HLA-B*, and only one tumor had *HLA-C* or *B2M* methylated (Fig. 4a). To further validate these results, we quantified HLA-A protein in 62 of the metastatic samples (Fig. 4b). We found a positive correlation of HLA-A protein with *HLA-A* mRNA (Fig. 4c) and with *HLA-B* mRNA but not *HLA-C* mRNA (Extended Data Fig. 6b). We also observed lower *HLA-A* mRNA expression in *HLA-A*-methylated tumors (Fig. 4d, left) and a near significant positive trend between HLA-A protein expression and *HLA-A* DNA methylation (Fig. 4d, right). DNA copy number analysis also demonstrated 23 samples from eight participants with focal deletions in this region, but in only 13 samples from three participants were these focal deletions near an *HLA* gene (<40 kb; Fig. 4e). From these 13 samples, only three tumors (two participants) had RNAseq data, and these focal deletions appeared nominally mutually exclusive from samples with *HLA-A* methylation (Fig. 4f). Following the same threshold applied to the *HLA-A* gene, three tumors from three different individuals had a focal deletion in the *B2M* gene (Fig. 4f). Other *HLA* class I-associated DNA methylation events appeared to be rare, except for *TAPBP*.

**Extended Data Fig. 7:**
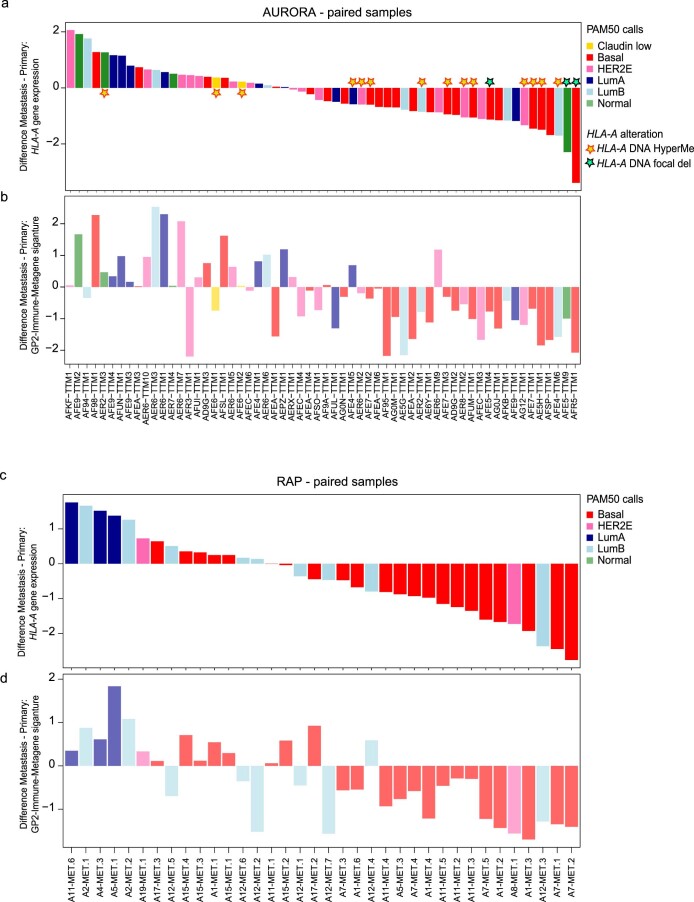
Difference in HLA-A and immune-signature expression between primary and metastatic tumors. **a**. Waterfall plot of AURORA cases showing the difference between primary (n = 36 tumors) and metastasis (n = 60 tumors) (Difference: Metastasis – Primary gene expression value) ordered from the highest (left) to the lowest (right) signature score for *HLA-A* mRNA expression (upper panel). The bottom panel shows the difference between primary and metastases for GP2-Immune-Metagene values. Yellow stars highlight *HLA-A* Hypermethylated cases and green stars highlight the samples with DNA *HLA-A* focal deletions. **b**. Waterfall plot of RAP cases showing the difference between primary (n = 12 tumors) and metastasis (n = 40 tumors) (Difference: Metastasis – Primary gene expression value) ordered from the highest (left) to the lowest (right) signature score for *HLA-A* mRNA expression (upper panel). The bottom panel shows the difference of primary versus metastases for GP2-Immune-Metagene values. Pairs with a Normal-like primary tumor were removed from the analysis. LumA, Luminal A; LumB, Luminal B.

Consistent with a functional role for these events, metastatic samples with *HLA-A* methylation or focal deletion had reduced mRNA of *HLA* genes and multiple immune signatures compared to their matched primaries (Fig. 4f). The *HLA-A*-, *HLA-B*- and *HLA-C*-altered samples also demonstrated a higher degree of *HLA-A*-predicted neoantigens (Fig. 4f). We also analyzed the relationship between *HLA-A* mRNA expression in primary tumors and paired metastases relative to immune signatures in the RAP dataset of 12 primary tumor–metastasis pairs and identified the same relationship of low *HLA-A* mRNA and low/lower immune cell gene expression features, which again was the most frequent in basal-like/TNBC (Extended Data Fig. 7a–d).

Interestingly, we noted a strong inverse association of *HLA-A*-predicted neoantigens with *HLA-A* gene expression, as opposed to *HLA-B* or *HLA-C*, in basal-like samples from both primary tumors and metastases (Extended Data Fig. 6c). In basal-like primary and metastatic tumors, those tumors with *HLA-A* alterations had significantly higher numbers of MHC class I-associated neoantigens, which was not driven by a higher tumor mutational burden (TMB; Fig. 4g); in particular, participant AER2 showed more than 50 times higher neoantigen load in primary tumors and liver metastases than observed in other participants. In this participant, *HLA-A* was methylated in primary and metastatic tumors, and *HLA-A* mRNA and immune signatures were even lower in the liver metastasis. By contrast, luminals and HER2E primary and metastatic tumors demonstrated higher TMB and MHC class I neoantigens in cases with MHC class I genetic or epigenetic alterations than in all other cases (Fig. 4g). Moreover, a general decrease in *HLA-A*, *HLA-B*, *HLA-C* and *B2M* gene expression was observed in basal-like samples with *HLA-A* genetic or epigenetic alterations (Fig. 4h). Taken together, these results point toward a high selective pressure on MHC class I-restricted neoantigens, CD8^+^ T cell-mediated immunity and MHC class I gene expression in basal-like BC. Of note, lower expression of MHC class I genes was observed in metastatic samples procured after increased lines of metastatic therapy (Fig. 4i), regardless of subtype.

We next tested the association of primary/metastasis-specific downregulation of an MHC class I metagene signature composed of a composite expression of *HLA-A*, *HLA-B*, *HLA-C*, *B2M*, *TAP1*, *TAP2* and *NLRC5* between metastasis and matched primary tumor according to intrinsic subtype. Across the AURORA and RAP datasets, only basal-like BCs demonstrated consistent and significant downregulation of the MHC class I metagene signature in metastatic disease (Fig. 4j). This downregulation was observed for *HLA-A*, *HLA-B* and *HLA-C* genes only in basal-like tumors (Extended Data Fig. 6d,e). Changes in gene expression for *HLA-A*, *HLA-B* and *HLA-C* genes were consistently altered within a given metastatic sample, supporting a common regulation of all three genes (Extended Data Fig. 6d,e).

To determine how antigen presentation via MHC class I expression and associated neoantigens may impact the tumor immune microenvironment, we performed CIBERSORTx^35^ deconvolution on RNAseq data in ‘relative mode’. We constructed a correlation matrix that was further analyzed by unsupervised hierarchical clustering. We observed four associated clusters of features, two of which reflected positive feature correlation patterns and two of which reflected negative feature correlation patterns (Extended Data Fig. 6f). The first positive cluster reflected associations of MHC class I neoantigens (specifically those with predicted binding affinity to HLA-A and HLA-C) with tumor-associated macrophages, regulatory T cells and γδ T cells. The second positive cluster showed enrichment of cytotoxic CD8^+^ T cells, memory-activated CD4^+^ T cells, B cells, dendritic cells (DCs) and inflammatory macrophages in high-MHC class I-expressing tumors, consistent with a more inflamed phenotype and intact antigen processing and presentation and adaptive immunity. Consistent with our prior finding that BCs with high MHC class I neoantigens appear to downregulate MHC class I gene expression, the first negative association cluster showed that tumors with more abundant neoantigens often were associated with poor DC cell activation hallmarks (negative cluster 1) and low expression of MHC class I genes (negative cluster 2).

**Extended Data Fig. 8:**
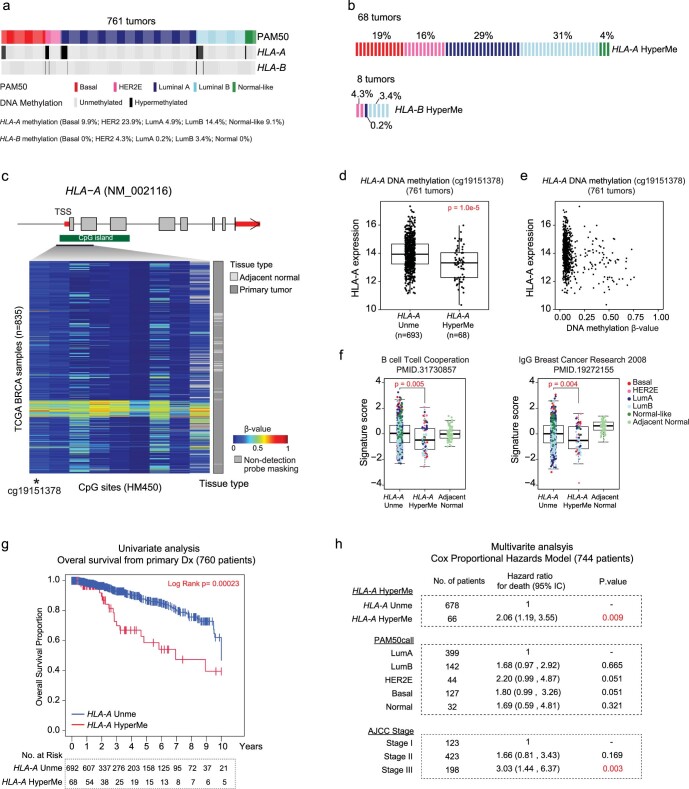
HLA-A methylated primary tumors and prognostic value of HLA-A in TCGA data. **a**. Oncoprint diagram depicting *HLA-A* and *HLA-B* methylated cases using 761 primary tumors of TCGA-BRCA dataset according to PAM50 molecular subtype. **b**. Proportion of each molecular subtype found in *HLA-A* (68) and *HLA-B* (8) methylated tumors. **c**. Hypermethylated CpG sites in *HLA-A* (9 CpG sites) using 761 TCGA primary breast tumors and 74 tumor-adjacent breast tissues (n = 835 samples). **d**. Boxplots of *HLA-A* mRNA gene expression levels according to DNA methylation status (n = 761 tumors). Comparisons between 2 paired groups were performed by *t*-test, two-sided. **e**. Scatter plot showing the correlation between *HLA-A* mRNA expression values and DNA methylation levels (ꞵ-values) (n = 761 tumors). **f**. Boxplots of gene expression signature B cell/T cell cooperation and IgG scores according to DNA methylation status in tumors and tumor-adjacent breast tissues in TCGA-BRCA (n = 761 tumors). Comparison between 2 groups was performed by ANOVA with post hoc Tukey’s test, one-sided. Statistically significant values are highlighted in red. Each mark represents the value of a single sample. **g**. Kaplan-Meier plots using the log-rank test of overall survival from primary tumors according to *HLA-A* methylation status (n = 760 tumors). **h**. Multivariable Cox proportional hazards analyses of TCGA BRCA patients for overall survival prediction using the covariates of *HLA-A* methylation status, PAM50 subtypes, and tumor stage (10 Stage IV patients were removed from the analysis) (n = 744). Hazard ratio (HR) = 1: no effect. HR < 1: reduction in hazard. HR > 1: increase in hazard. Statistically significant values are highlighted in red. All Box-and-whisker plots display the median value on each bar, showing the lower and upper quartile range of the data (Q1 to Q3) and data outliers. The whiskers represent the lines from the minimum value to Q1 and Q3 to the maximum value. Unme, unmethlylated; HyperMe, hypermethylated. LumA, Luminal A; LumB, Luminal B.

Given the finding of *HLA-A* loss in the metastatic setting, we also sought to determine whether this might occur in early-stage disease and how frequently by evaluating TCGA-BRCA data that contain RNAseq, DNA-sequencing and DNA methylation data^36^. Of 761 TCGA-BRCA tumors tested, 68 showed methylation of *HLA-A*, and 8 showed methylation of *HLA-B* (Extended Data Fig. 8a–c). Primary tumor *HLA-A* methylation was associated with lower *HLA-A* mRNA levels and lower expression of multiple adaptive immunity signatures (Extended Data Fig. 8d–f). Importantly, tumors with *HLA-A* methylation showed worse survival outcome, even in multivariate analyses adjusting for stage and PAM50 subtype (Extended Data Fig. 8g,h).

### Epigenetic suppression of cell adhesion in metastases

**Fig. 5:**
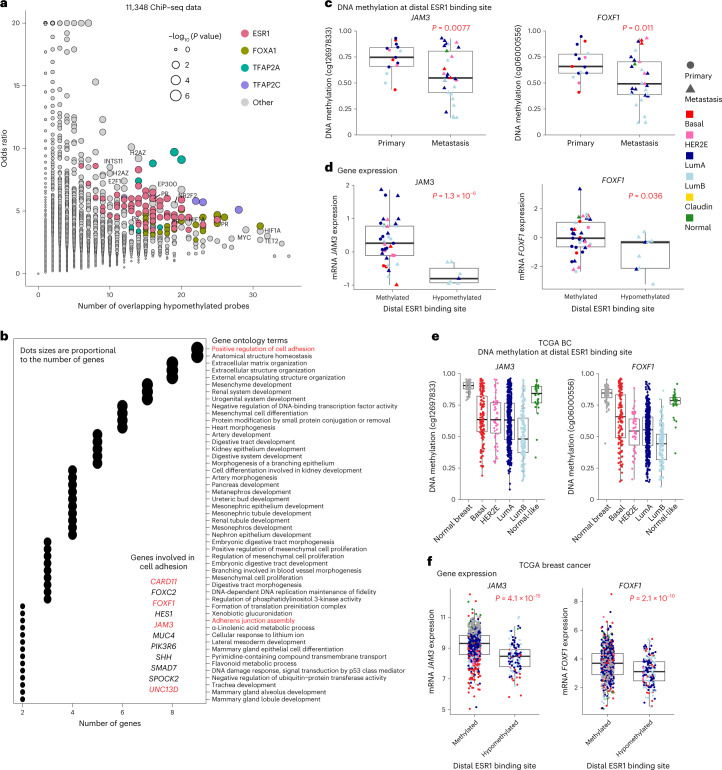
Metastatic tumor-associated DNA hypomethylation at distal enhancer elements. **a**, Analysis of DNA binding proteins at the significantly hypomethylated CpG sites. Each dot represents 1 of the 11,348 ChiP–seq datasets analyzed. The *y* axis represents the odds ratio of enrichment, and the *x* axis represents the number of significant CpGs overlapping protein binding sites. The size of the dot denotes the statistical significance of the enrichment (Fisher’s exact test); HR, hormone receptor. **b**, GO analysis of putative target genes for the hypomethylated *ESR1* or *FOXA1* distal binding sites. Shown are the top 50 GO terms based on the *P* values from the Fisher’s exact test. Dot sizes are proportional to the number of genes. Red text highlights cell adhesion GO terms and genes of interest. **c**, Analysis of putative enhancer target genes involved in the regulation of cell adhesion in ER^+^ tumors. A comparison of distal element DNA methylation between primary tumors (*n* = 15 tumors) and metastases (*n* = 19 tumors) in ER^+^ tumors is shown. **d**, Gene expression between methylated (*β* value of ≥0.4) and unmethylated (*β* value of <0.4) ER^+^ tumors. The *P* values of **c** and **d** were calculated using Welch’s two-sample *t*-test (two sided). **e**,**f**, Analysis of distal element DNA hypomethylation (**e**) and putative target gene expression (**f**) in TCGA BC data (*n* = 835 tumors, 761 primary tumors and 74 adjacent normal tissue). Normal breast tissue samples are indicated in dark gray, and tumor samples are color coded by the PAM50 molecular subtype. The samples were identified as either methylated or unmethylated using a *β* value threshold of 0.4. The *P* values were calculated using Welch’s two-sample *t*-test (two sided). All box and whisker plots display the median value on each bar, showing the lower and upper quartile range of the data (Q1 to Q3) and data outliers. The whiskers represent the lines from the minimum value to Q1 and Q3 to the maximum value. LumA, Luminal A; LumB, Luminal B; Claudin, Claudin-low.

**Extended Data Fig. 9:**
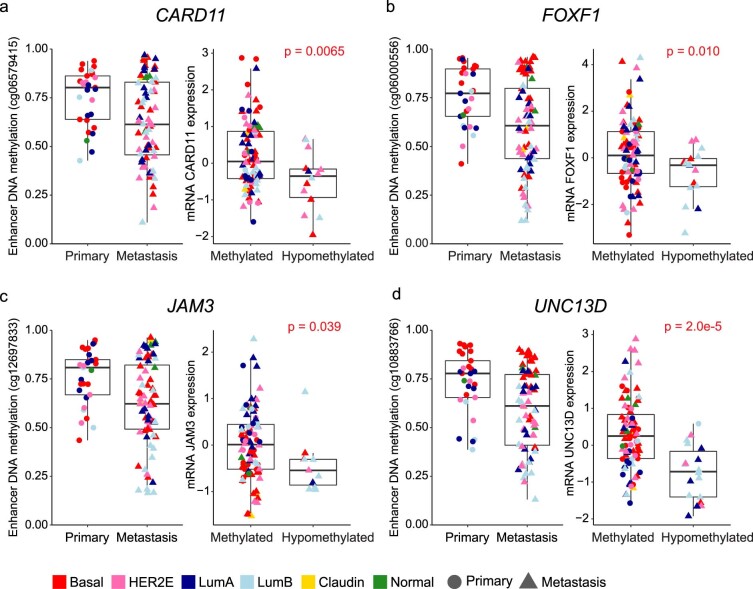
Metastatic tumor-associated DNA hypomethylation at distal enhancer elements. **a-d**. Analysis of putative enhancer target genes involved in the regulation of cell adhesion. For each gene, a comparison of distal element DNA methylation between 29 primary and 72 metastatic tumors is shown on the left, and putative target gene expression between methylated (β value ≥ 0.4) vs. unmethylated (β value of < 0.4) tumors is shown on the right. The *p values* were calculated using Welch’s two-sample *t*-test, two-sided. LumA, Luminal A; LumB, Luminal B; Claudin, Claudin-low.

We conducted a systematic analysis of DNA methylation changes associated with metastasis to uncover additional genes affected by an epigenetic mechanism. Cellular composition has a profound impact on DNA methylation profiles; thus, different metastatic sites could produce false-positive results through contaminating stromal DNA methylation signals. We circumvented this metastatic site contamination problem by screening for loss of methylation in metastatic tumors at *cis*-regulatory elements that are consistently methylated in normal tissues representing the metastatic target tissues. We selected 19,607 CpG sites in distal enhancer-like elements defined by the ENCODE project^37^ that are constitutively methylated in eight normal tissue types. Statistical testing analyses comparing primary tumors to metastases identified 123 CpG sites that were significantly hypomethylated in metastatic tumors compared to their matched primaries. Using 11,348 chromatin immunoprecipitation with sequencing (ChIP–seq) datasets, we found a significant overrepresentation of 47 DNA binding sites for 21 proteins at the 123 hypomethylated CpG sites (Fig. 5a). Proteins involved in estrogen signaling dominated binding at these hypomethylated CpGs, including those encoded by *ESR1*, *FOXA1*, *TFAP2A* and *TFAP2C*, consistent with other reports of estrogen signaling in BC progression^38,39^. We further investigated the distal elements bound by ESR1 and FOXA1 by performing Gene Ontology (GO) enrichment analysis of putative target genes regulated by these elements (Methods and Fig. 5b). We found that genes involved in the regulation of cell adhesion are frequently represented among the target genes (Fig. 5b). However, surprisingly, we found that distal element hypomethylation is significantly associated with reduced expression of these associated genes, suggestive of negative regulation of these genes by estrogen signaling when analyzing individuals with ER^+^ BC only (Fig. 5c,d) or even when using all individuals (Extended Data Fig. 9a–d). We confirmed the significant association between distal element hypomethylation and reduced expression of *JAM3* and *FOXF1* in TCGA (Fig. 5e,f).

**Extended Data Fig. 10:**
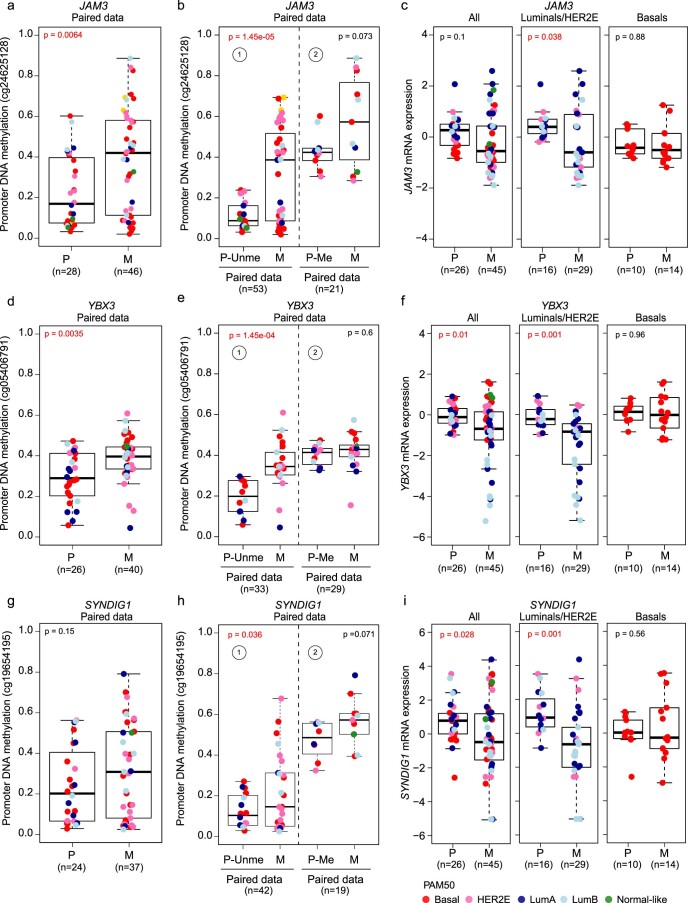
DNA methylation alterations associated with metastatic tumors. **a-i**. Analysis of metastasis-associated promoter DNA hypermethylation of three genes (*JAM3, YBX3 and SYNDIG1*) encoding components of tight junctions or regulation of adhesion molecules. For each gene, a comparison of promoter CpG DNA methylation between primary and metastatic tumors is shown on the left (a, d, g), a second comparison of promoter CpG DNA methylation between ① unmethylated primaries (β-value of <0.3) and their paired metastasis and ② methylated primaries (β-value of > 0.3) with their paired metastasis (b, c, e) is shown in the middle, and a third comparison of gene expression between primary and metastatic tumors based on all samples (All), Luminal A-B and HER2E only (luminals/HER2E), and basal-like subtype only (basals) is shown on the right (c, f, i). LumA, Luminal A; LumB, Luminal B; P, primary; M, metastasis; P-Unme, Unmethylated primary; P-Me, Methylated primary.

We conducted a similar screen for gain of methylation at promoters by selecting CpG sites that are constitutively unmethylated in normal tissues representing the metastatic target tissues. We identified metastasis-associated promoter DNA hypermethylation of three genes (*JAM3*, *YBX3* and *SYNDG1*), one of which was also identified in the distal element DNA hypomethylation analysis (Extended Data Fig. 10, left and middle). Gene expression of all three genes was significantly lower in metastatic tumors than in the matched primaries, and this observation was more pronounced in HER2E or luminal subtypes (Extended Data Fig. 10c,f,i).

### Clonal evolution and subtype switching

**Fig. 6:**
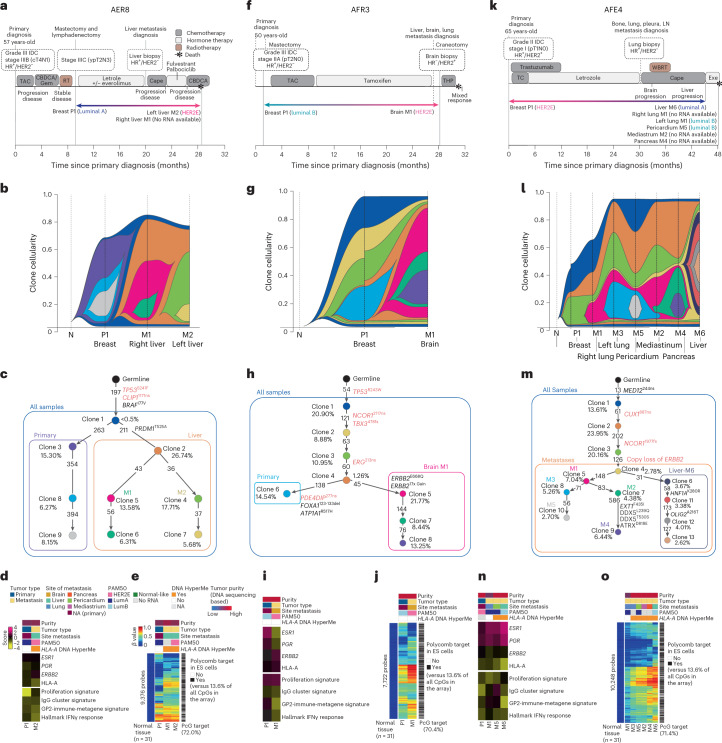
Multiomics participant characterization of individual AURORA cases. **a**–**o**, Timeline of participant clinical history (**a**, **f** and **k**), clonal structure (**b**, **g** and **l**), clonal evolution (**c**, **h** and **m**) and transcriptome (**d**, **i** and **n**) and methylome description (**e**, **j** and **o**) of participants AER8 (**a**, **b**, **c**, **d** and **e**), AFR3 (**f**, **g**, **h**, **i** and **j**) and AFE4 (**k**, **l**, **m**, **n** and **o**). Transcriptome data reflect gene expression values, and gene expression signatures were calculated using normalized RNAseq data; LumA, Luminal A; LumB, Luminal B; P, primary; M, metastasis; N, AQ21normal; LN, lymph node; R. Lung, right lung; L. Lung, left lung; R. Liver, right liver; L. Liver, left liver; M, metastasis; ES, embryonic stem. PGR, progesterone; ESR1, estrogen receptor; TAC, docetaxel (Taxotere), doxorubicin hydrochloride (Adriamycin), and cyclophosphamide; CBDCA, carboplatin; Gem, gemcitabine; RT, radiation therapy; Cape, capecitabine; THP, docetaxel, trastuzumab, and pertuzumab; WBRT, whole brain radiation therapy.

Many publications have studied DNA-based clonal evolution in longitudinal samples and in response to therapeutic selection^40,41^. We focused here on three cases that showed gene expression-based subtype switching to address the question of whether this change in expression phenotype was accompanied by DNA clonality changes (Fig. 6a–o). Participant AER8 was diagnosed with an ER^+^/progesterone receptor (PR)^+^/HER2^–^ LumA subtype primary tumor and received neoadjuvant chemotherapy and adjuvant endocrine therapy plus everolimus; participant AER8 was diagnosed with liver metastases after ~20 months of treatment, received an additional three lines of therapy and succumbed to disease, at which time biopsies of several metastatic lesions were obtained (Fig. 6a). The two assayed liver metastases were of the same clonal lineage (orange), which was distinct from the dominant clonal lineage of the primary (purple; Fig. 6b,c), a finding also supported by the DNA hypermethylation profiles (Fig. 6e). Metastasis M2 was assayed by RNAseq and showed a subtype switch to HER2E (yet remained clinically HER2^–^), with an increase in proliferation signature and a decrease in *HLA-A* mRNA levels and immune cell features (Fig. 6d). Acquisition of the HER2E subtype in the absence of gain of *HER2* amplification in metastatic samples has been reported^3,22,42^.

A second example of subtype switching was participant AFR3, who was diagnosed with an ER^+^/PR^+^/HER2^–^ LumA BC. Participant AFR3 was treated with chemotherapy then endocrine therapy and progressed with multiple metastases, of which the brain metastasis was surgically removed (Fig. 6f). The brain specimen showed a dramatic change to ER^–^/PR^–^/HER2^+^, and gene expression analysis confirmed an increase of *HER2* expression and a subtype switch to HER2E (Fig. 6f), with a concomitant DNA clonality change that included the acquisition of copy amplification of the *HER2* region and an *ERBB2*/*HER2* E668Q activating mutation (Fig. 6g,h). This DNA clonal change was also reflected in the DNA hypermethylation landscape (Fig. 6j) and was associated with a downregulation of *ESR1* and *PGR* and upregulation of *ERBB2* mRNA (Fig. 6i).

In contrast to participant AFR3 whose BC switched to the HER2E subtype, likely due to an acquired *HER2* amplification, participant AFE4 showed a reverse trend. Namely, this participant presented with an ER^+^/PR^+^/HER2^+^ BC (HER2E expression subtype), where it was noted that the clinical HER2 immunohistochemistry (IHC) result was 2+ and fluorescence in situ hybridization (FISH) inconclusive but was HERmark assay positive. After 30 months of trastuzumab, tumor progression was documented, a lung biopsy was obtained, and the clinical receptor status was remeasured, indicating an ER^+^/HER2^–^ status. Additional treatments were given; however, the tumor progressed, and the participant died 18 months later. At autopsy, multiple metastatic tumor specimens were obtained (Fig. 6k). Interestingly, the three metastatic specimens assayed by RNAseq showed subtype switches to LumA or LumB, DNA clonality changes and loss of *HER2* amplification (Fig. 6l,m), while *HER2* mRNA levels were slightly decreased (Fig. 6n). DNA methylation features largely agreed with the DNA clonal evolution except for right lung metastasis (M1) that presented with the lowest DNA tumor purity score (Fig. 6o). Interestingly, liver metastasis (M6) was the most clonally distinct metastasis (as it is shown by clonal evolution history and DNA methylation), showing a subtype switch to LumA and the lowest levels of *HLA-A* expression and immune-related signatures compared to the other metastases.

## Discussion

Established metastatic tumors are challenging to treat, and their biology is complex. Overall, when primary tumors are compared to their matched metastases, the dominant genomic patterns seen in the primary tumors tend to be maintained in the metastases; however, significant differences have been identified that may contribute to the poor prognosis associated with MBC. In performing multiplatform analyses of primary tumors versus metastases, we discovered several patterns that may explain some metastatic tumor behaviors, including events derived from epigenetic, genomic and transcriptomic evolution.

A key epigenetic mechanism identified here was DNA methylation of *HLA-A* and *HLA-A* small focal deletions, typically in basal-like/TNBC metastatic disease, leading to lower expression of *HLA-A* and associated lowered expression of immune cell features. Alterations in *HLA-A* have also been described using loss of heterozygosity (LOH) analyses in BCs^43^ and by simply lower mRNA^44^. Here, we show a lower expression of *HLA-A* in those TCGA primary cancers with DNA methylation and, when observed, was linked to lower immune cell features and a worse overall survival. These findings provide a molecular explanation for the loss of immune cell features in some metastatic tumors, which has potential therapeutic implications. One such implication is that ICIs may have little effect on these *HLA-A*-low tumors, as these cannot be recognized by CD8^+^ T cells (noting these *HLA-A*-methylated tumors tend to have high neoantigen burdens). These results also suggest a biomarker-driven therapeutic approach wherein *HLA-A* DNA-methylated tumors (that is, the biomarker) could be targeted with DNA demethylating drugs in combination with ICIs^45^.

Changes in the somatic genetics of metastatic breast tumors are well documented^2,3^, and here we extend the changes seen in metastatic tumors into the epigenetic landscape. Gene expression subtype discordance between primary and metastatic tumors has been previously described^22,46,47^, and the AURORA study here identified similar findings. Namely, in one of three individuals with BC, we identified a gene expression tumor subtype switch, which was especially frequent in individuals with luminal/ER^+^ BC. In addition to possible epigenetic changes in tumor cells, RNAseq analysis of multiple immune cell signatures showed dramatic differences simply according to site of metastasis. It is already appreciated that the brain is an immune-privileged site^48^, and our results confirm this finding. There is also growing evidence that the liver is similarly immune privileged^49^, and our results confirm low immune cell features in liver metastases. Using this unique resource, we found that in 9 of 14 individuals with multiple metastases, liver metastases had the lowest immune cell features of any synchronous site of metastasis. These comparative metastatic tissue site findings have clinical implications because the liver is a commonly biopsied site for metastatic evaluation, and our data suggest that liver metastases are more likely to have low immune cell features, which may bias assay results of immune therapy biomarker positivity.

Interestingly, we discovered through systematic screening for metastasis-associated DNA methylation changes mechanisms leading to downregulation of *JAM3* expression in metastatic tumors, namely DNA hypomethylation at a distal ESR1 binding site and DNA hypermethylation of the gene promoter. Notably, it has been reported that *JAM2* overexpression (a second JAM family member) in BC cell lines blocks invasion and migration^50^, *JAM3* is silenced by DNA hypermethylation in colorectal cancers, and *JAM3* suppression promotes migration^51^. In addition, a causal interaction between DNA methylation and ER-mediated repression of gene expression has been previously reported^52^, and our finding that multiple genes regulating cell adhesion appearing to be negatively regulated by estrogen signaling may have functional consequences for progression to metastasis. This is consistent with prior reports of estrogen-mediated downregulation of E-cadherin in BC cells^53^.

Finally, our three examples of clonal evolution highlight DNA clonality shifts coincident with gene expression-based subtype changes. In participant AER8, the clonal shift and altered expression subtype did not include any new actionable mutations, which may represent the most common finding with respect to changes in DNA-based actionable mutations in the metastatic setting^54^. In participant AFR3, an actionable variant was identified (that is, gain of *HER2*), and trastuzumab therapy was given, although the tumor progressed. Participant AFE4 highlights yet another challenge of precision medicine wherein an actionable DNA-based feature is identified and targeted (that is, *HER2* amplification), yet the tumor eventually evades the treatment by deleting the therapeutic target. Each of these participants illustrates a third clinical impact of this study, which is if medically possible, biopsy and characterize the metastatic disease as it has likely changed relative to the primary tumor.

There are limitations to this study. The first challenge was that the sample size was likely underpowered to find somatic mutation frequency differences. The second challenge was the integration of data from FF specimens with data from FFPE specimens. The third challenge was that participants received multiple adjuvant and/or metastatic treatments, and we were not able to evaluate the treatment effects (noting each participant had an average of three lines of therapy). Nonetheless, we identified many multiplatform-supported findings concerning tumor clonal evolution and immune evasion that are common in MBCs. This multiplatform genomic data resource of metastatic disease presented here is highly complementary to the TCGA resource of primary disease^36,55,56^ and has already begun to illuminate the molecular landscape of MBC.

## Methods

### Clinical summary

All research involving human tumor tissues was reviewed and approved by the appropriate Institutional Review Board of Research at Baylor College of Medicine, Dana Farber Cancer Institute, Duke University, Georgetown University Medical Center, Indiana University, Mayo Clinic, Memorial Sloan Kettering Cancer Center, University of Pittsburgh and UNC at Chapel Hill, and the studies were performed in accordance with recognized ethical guidelines. We obtained a waiver of written informed consent for some participants for the use of their biological specimens, and in other protocols, we obtained informed consent for the research procedures. Samples from a total of 55 female participants with MBC were the final dataset of the AURORA US cohort. Of these 55 participants, 10 (18%) were of African American descent, and 4 (7%) were of Hispanic ethnicity. The median age at initial BC diagnosis was 49 years (range: 25–76). Forty-nine participants (89%) initially presented with stage I to stage III BC, of which 19 (38%) received neoadjuvant systemic therapy, and 6 (10%) presented with de novo metastatic disease. In the metastatic setting, participants received a median of three lines of systemic therapy (range: 0–20). Metastatic samples from a total of 20 participants were collected at autopsy. Additional clinicopathologic features are displayed in Supplementary Table 1.

### Pathology review

Pathology quality control (QC) was performed on each tumor specimen and normal tissue specimen as an initial QC step. Hematoxylin and eosin-stained sections from each sample were subjected to independent pathology review to confirm that the tumor specimen was histologically consistent to the reported histology. The percent tumor nuclei, percent necrosis and other pathology annotations were also assessed. Tumor samples with ≥30% tumor nuclei and normal tissue with 0% tumor nuclei were submitted for nucleic acid extraction. All hematoxylin and eosin images are also available and part of this data resource.

### AURORA sample acquisition and biospecimen processing

RNA and DNA were extracted from frozen tissues using a modification of the AllPrep DNA/RNA kit (Qiagen). The flow-through from the Qiagen DNA column was processed using a mirVana miRNA isolation kit (Ambion). RNA and DNA were extracted from FFPE solid tissues using a modification of the AllPrep DNA/RNA FFPE kit (Qiagen). The flow-through from the Qiagen DNA column was processed using a mirVana miRNA isolation kit (Ambion). For cases in which whole blood or blood derivatives were received, DNA was extracted from blood using the QiaAmp DNA blood midi kit (Qiagen). RNA samples were quantified by measuring absorbance at 260 nm with a UV spectrophotometer, and DNA was quantified by PicoGreen assay. DNA specimens were resolved by 1% agarose gel electrophoresis to confirm high-molecular-weight fragments. A custom Sequenom single-nucleotide polymorphism panel or the AmpFISTR Identifiler (Applied Biosystems) was used to verify that tumor DNA and germline DNA representing a case were derived from the same participant. RNA was analyzed via the RNA6000 Nano assay (Agilent) for determination of an RNA integrity number. Only cases yielding a minimum of 250 ng of tumor DNA, 500 ng of tumor RNA and 250 ng of germline DNA were included in this study. A minimum of one QC-qualified tumor sample and one QC-qualified normal tissue sample were required for a case to become part of the study (*n* = 55 total cases).

### RNAseq, gene expression data values and normalization

Gene expression profiles from primary and metastatic tumors for the AURORA dataset were generated by RNAseq using an Illumina HiSeq and an rRNA depletion method. Briefly, 300–500 ng of total RNA was converted to RNAseq libraries using the TruSeq Stranded Total RNA Library Prep kit with Ribo-Zero Gold (Illumina) and sequenced on an Illumina HiSeq 2500 using a 2 × 50 base pair (bp) configuration. QC-passed reads were aligned to the human reference CGRh38/hg38 genome using STAR v.2.7.6a. Transcript abundance estimates for each sample were performed using Salmon v. 1.4.0, an expectation maximization algorithm using the University of California Santa Cruz gene definitions. Raw read counts for all RNAseq samples were normalized to a fixed upper quartile (UQN). The raw reads files are available in dbGAP (phs002622.v1.p1).

### Gene expression analysis of RNAseq data and batch effect adjustments

RNAseq UQN gene counts from 123 primary and metastatic tumors comprised of 35 FFPE and 88 FF RNA-sequenced tumor data were log_2_ transformed, genes were filtered for those expressed in 70% of samples, and zeros were returned to the empty values. To improve the batch effect between the two data types (that is, FFPE and FF), we merged a second dataset of 101 paired primary and metastatic tumors (UNC RAP cohort) comprised of 20 FFPE and 81 FF sequenced tumors. This second dataset was partially previously published in 2018 (ref. 23), but some new samples were added and sequenced for the present work, and many of the published samples were resequenced here using the rRNA depletion method (dbGAP phs002429). The RAP101 samples of the present work were created with the same RNA extraction, library preparation and sequencing protocol as the AURORA samples and represent a second dataset of FFPE and FF samples that increases our sample size for adjustments of FFPE versus FF effects. The clinical information of the RAP101 dataset is found in Supplementary Table 2.

To address this systematic effect, we merged the raw read counts for all RNAseq samples of the previously mentioned RAP101 dataset with 123 samples of the AURORA study (level 1 data). These counts were normalized using DESeq2-normalized counts (median of ratios method)^57^. Briefly, we created a DESeq2Dataset object and generated size factors using the estimateSizeFactors() function. Next, to retrieve the normalized counts matrix, we used the counts() function and added the argument normalized=TRUE. After generating the normalized count matrix, genes with an average expression lower than 10 were filtered from the dataset. RNAseq-normalized gene counts from the 224 dataset were log_2_ transformed (level 2 data). Next, we used the removeBatchEffect() function from the limma R package^58^, including both batches in the formula. Last, we subtracted only the 123 samples from the AURORA study and used this normalized, log_2_-transformed and batch-corrected dataset for further RNAseq gene expression analysis (level 3 data).

To minimize false-positive results due to the normal tissue contamination generated by normal brain (*n* = 10), liver (*n* = 8) or lung tissue (*n* = 7), the most common sites of metastasis in this study, we removed those genes whose expression was solely coming from these three tissue sites. Specifically, we used statistical testing to determine normal brain, liver and lung signatures by comparing each normal tissue to normal breast tissue (*n* = 5; Supplementary Table 3; dbGAP accession number for AURORA phs002622.v1.p1 and for RAP and 9830 phs002429). This normal tissue dataset was also created using the same RNA extraction, library preparation and sequencing protocols. From normalized, filtered and median-centered counts, we performed linear model (LM) regression using lme4 (ref. 59) and lmerTest^60^ R packages given the formula, fit = lm(genes ~ normal site of metastasis/breast normal), and *P* values were adjusted for multiple comparisons using the Benjamini–Hochberg approach^61,62^. We obtained the most significant upregulated genes in each normal tissue (FDR < 0.00001) by comparing each normal tissue to normal breast tissue (brain versus breast, liver versus breast and lung versus breast); we merged these three lists and identified 1,900 genes as the distinctive upregulated genes of our ‘normal tissue signature’. To build a second signature characteristic of breast primary tumors, we did a second LM analysis between the 46 primary tumors from the AURORA study and the 5 normal breast tissue samples from the above-mentioned normal tissue cohort, and we obtained 833 significant upregulated genes (FDR < 0.01). Some of these genes were also present in the ‘normal tissue signature’, and thus we removed these common 449 genes from the ‘normal tissue signature’ list, considering these genes not unique to normal tissues but also important markers for primary tumors in the AURORA cohort. Finally, the remaining 1,451 genes of the ‘normal tissue signature’ (Supplementary Table 3) were removed from the original normalized and batch-corrected gene expression data matrix of the 123 AURORA cohort samples (referred to as the normalized, log_2_-transformed, batch-corrected and normal-adjusted data or level 4 RNAseq data).

#### PAM50 subtype classification

To better maintain methods with past intrinsic subtyping methods^24^, for PAM50 subtype classification assignments, we normalized the RNAseq data in a different way than described immediately above that is based on within-dataset row and column standardizations. Briefly, RNAseq-normalized gene counts from 123 primary and metastatic tumors comprised of 35 FFPE and 88 FF RNA-sequenced tumor data were log_2_ transformed, genes were filtered for those expressed in 70% of samples, and zeros were returned to the empty values. To address the FFPE versus FF effects, we again used the AURORA and RAP101 datasets as described above and made an adjustment for FFPE versus FF. Namely, using only common genes between both datasets, we merged, row median centered and column standardized FFPE and FF groups separately, where each gene was a row, and each sample was a column. Next, we subtracted only the FFPE and FF normalized batches from the AURORA study and used these values for receiver operating characteristic (ROC) curve and Youden cutoff analysis for ER, PR and HER2 status comparisons, which provide external validation that the adjustments do not adversely affect the gene expression data using tests of correlation to the external clinical standards.

For PAM50 subtype classification, we applied a HER2/ER subgroup-specific gene-centering method as described in the supplemental methods of Fernandez-Martinez et al.^24^. For applying this subgroup-specific gene-centering method, we need the IHC status for all samples assayed by RNAseq. Six percent of primary tumors and 39% of metastatic samples did not have HER2 IHC information, and 38% of metastatic samples were missing ER status. ‘Profiled Primary ER/HER2/PR’ columns of Supplementary Table 2 were used for this analysis. We again used ROC curve and Youden cutoff values for inferring protein clinical status using *ESR1* and *ERBB2* gene expression data from all tumors, and we assigned ER and HER2 clinical status to those samples that had missing clinical values using the mRNA surrogates. The ROC curve analysis showed a value of 0.92 for ER status by *ESR1* mRNA and of 0.87 for HER2 status using *ERBB2* mRNA. The new RNAseq-inferred ER/PR/HER2 protein status was used for the subgroup-specific gene-centering method (inferred ER/PR/HER2 column of Supplementary Table 2). Finally, the gene expression values of the PAM50 genes using the UQN gene counts were then normalized, and the PAM50 predictor^63^ was applied using the provided centroids to assign subtype calls using correlation values for all primary tumors and metastases (Supplementary Table 2).

#### Gene expression signatures

For each batch-corrected and adjusted for normal tissue gene expression dataset/subset (level 4 RNAseq data), we applied a collection of 747 gene expression modules (Supplementary Table 3), representing multiple biological pathways and cell types, to all primary and metastatic tumors^22,31,64^.

Finally, we developed an immune metagene signature named ‘GP2-immune-metagene’, a signature that we developed to capture immune cell features as derived from the AURORA dataset. Briefly, we used TCGA gene expression data to calculate all 747 module scores, which was then used for hierarchical clustering analysis, and the resulting clusters of modules were tested for significance of these groups of modules using SigClust^65^. Fifty-six clusters with a *P* value of <0.001 were identified, and 16 immune-related signatures from cluster 51 were grouped as a new ‘immune meta-signature’ named the GP2-immune-metagene signature (Supplementary Table 3); included within this group of immune clusters were signatures of T cells, B cells, macrophages and DCs. Next, using our previously calculated 747 gene expression module scores from the AURORA dataset, we selected the 16 immune-related signatures and calculated the means of these 16 signatures for each participant and called this newly derived signature ‘GP2-immune-metagene’.

#### Merging UNC RAP, GEICAM and AURORA cohorts (RNAseq only)

To study metastasis in an organ-specific manner, we increased the number of the most common sites of metastasis (lung, liver and brain) creating a larger dataset. We merged the data of the AURORA and RAP101 cohorts and 204 samples of the GEICAM cohort^22^. Sample acquisition and biospecimen processing followed the same protocols as the AURORA cohort and were also sequenced at UNC through the High-Throughput Sequencing Facility.

Next, we corrected the technical bias detected between the gene expression of 259 FFPE and 169 FF samples from 176 primary and 411 metastatic tumors (428 tumors in total) following the same scheme as for correction of AURORA batch effects (including FFPE and FF as batches in the formula). To minimize the false-positive results due to the normal tissue contamination, we proceeded as we did in the AURORA dataset, 1,451 genes of the ‘normal tissue signature’ (Supplementary Table 3) were removed from the data matrix of the 428 AURORA–RAP–GEICAM cohort. From this merged set that is already batch corrected and adjusted by normal tissue, we subtracted samples from the RAP cohort that were exact duplicates or coming from the same original tissue also used in the AURORA cohort; this removed 20 of the RAP101 samples. The final cohort of 82 tumors is listed in Supplementary Table 2, sheet 5 (RAP study), column name ‘Freeze cohort_RAP’. This yielded a final cohort of 409 tumors in total (155 participants with 155 primaries and 211 paired metastases and 11 unpaired primaries and 32 unpaired metastases), each summarized in Supplementary Table 2.

Next on the three-dataset combined data matrix, we calculated the gene signature score for each module as described before, and we performed a linear mixed model (LMM) using lmerTest^60^ and lme4 R packages to identify significantly changed modules between metastatic and primary tumors. In the LM, we included the term ‘patient’ as random effect or confounding variable, fit = lmer(genes ~ met/prim + (1|patient), using all the primary and metastatic tumors except the primaries identified as post-treatment primaries (participants who received neoadjuvant therapy before primary tumor collection). To avoid the possible confounding factor of intrinsic molecular subtype in the subsequent analysis, we divided tumors into two datasets based on the subtype of the primary tumor from each pair: a ‘luminal set’ comprising all LumA, LumB and HER2E subtype participants and a ‘basal-like set’ containing basal-like subtype participants only; samples called normal-like in the primary or metastatic tumors or post-treatment primary tumors were removed from the analysis (column ‘Groups PAM50 Gene Expression Analysis’ from Supplementary Table 2). To identify significantly changed modules between brain or liver and their corresponding primary tumors only, the studied sites of metastasis versus the corresponding primary pair were compared using the same lmer function. The significantly differentially expressed modules (*q* < 0.05) were hierarchically clustered using the ComplexHeatmap R package. HeatmapAnnotation and Heatmap functions were used to show the heatmap that was previously row ordered by primary and metastatic tumors and column ordered by estimates or *β* values. Differential gene expression module analysis in the merged AURORA–RAP–GEICAM set was performed in the same way as AURORA only. Multimetastatic samples derived from AURORA and RAP and single primary–tumor pairs derived from GEICAM with PAM50 classification of normal-like in primary or metastatic tumors and post-treatment primary tumors were removed from the analysis. For the comparisons between site of metastasis using the merged set, we performed SAM^66^ analysis of the list of 747 gene expression modules between 46 liver metastases and 18 brain metastases, 46 liver metastases and 24 lung metastases, 46 liver metastases and 35 lymph node metastases, 18 brain metastases and 35 lymph node metastases and 24 lung metastases and 18 brain metastases (FDR = 0; Supplementary Table 3).

### Statistics and reproducibility

No statistical method was used to predetermine the sample size that was limited by the size of the samples provided and successfully assayed for this study.

For LMM/linear mixed-effects model and LM analyses between primary and metastatic tumors, the lmerTest^60^ R package summary includes a coefficient table with estimates and *P* values for *t*-statistics using Satterthwaite’s method. These *P* values were adjusted for multiple comparisons using the Benjamini–Hochberg approach^61,62^. Non-parametric, two-sided exact tests were used to make comparisons. A *t*-test (two sided) was used for comparisons between two groups, and a Mann–Whitney *U*-test was used when the dependent variable was either ordinal or continuous but not normally distributed. A paired *t*-test (two sided) was used for analyzing repeated measures within the same groups. Comparisons between more than two groups were performed by analysis of variance (ANOVA) with a post hoc Tukey test (one sided). Exact *P* values were provided whenever possible. The strength of correlations was measured using the Pearson (*P*) or Spearman (*ρ*) correlation coefficient and the probability of observing a correlation with the corresponding *P* values. Clinical, RNAseq, DNA-sequencing and DNAme analyses were performed using RStudio version 1.4.1103 (http://cran.r-project.org), GraphPad Prism 9.0 software and/or Microsoft Excel (version 2210 build 16.0.15726.20070). More details about each particular platform analysis are found in each methodology section. No randomization or blinding was done in the data collection or analyses. No data points were excluded from the analyses unless is specified otherwise.

### TCGA RNAseq data

We analyzed the BC dataset from TCGA project profiled using the Illumina HiSeq system. We included 1,095 primary tumors and 97 adjacent non-malignant tissues for developing the immune signature named ‘GP2-immune-metagene’ and 761 primary tumors and 74 adjacent non-malignant tissues for the *HLA-A*-methylated primary tumor analysis and prognostic value of HLA-A. TCGA files were downloaded from Broad GDAC Firehose (Supplementary Table 7).

### HLA-A immunofluorescence staining

FFPE tissue was sectioned at 4 µm and stained with a CK/HLA-A assay developed and optimized at Vanderbilt University Medical Center using tyramine signal amplification for increased antigen sensitivity. Sections were deparaffinized. Antigen retrieval was performed with citrate buffer at pH 6. Endogen peroxidase was blocked with hydrogen peroxide, and protein block was applied. Sections were then incubated with the first primary antibody, pan-cytokeratin (pan-CK) AE1/AE3 Biocare, at 1:1,600 overnight at 4 °C, followed by incubation with the secondary antibody conjugated with horseradish peroxidase. TSA reagent was applied according to manufacturer’s recommendations. After washing, antigen retrieval and protein block steps, the second primary antibody, HLA-A C6 Santa Cruz at 1:1,300, was incubated overnight as described. Counterstaining was performed with DAPI for nuclei identification. Tonsil and placenta tissue were used as positive- and negative-control tissues.

Whole-slide images were digitally acquired using an AxioScan Z1 slide scanner (Carl Zeiss) at ×20 magnification. Automated quantification was performed via a pathologist-supervised machine learning algorithm using QuPath software. Cell segmentation was determined on DAPI. Object classifiers were trained on annotated training regions from control tissue and tumor samples to define cellular phenotypes. Tumor cells were defined by pan-CK expression and subcellular characteristics. Once the algorithm was performing at a satisfactory level, it was used for batch analysis. For cases with low, heterogenous or null CK expression in which the classifier performance was not optimal, tumor areas were manually annotated. Out-of-focus areas, tissue folds, necrosis, normal breast and in situ carcinoma were excluded from the analysis. Single-cell data were exported from QuPath, and mean HLA-A intensity on tumor cells was further calculated in R.

### Array-based DNA methylation assay

DNA methylation was evaluated using the Illumina HumanMethylationEPIC (EPIC) array. The EPIC platform analyzes the DNA methylation status of up to 863,904 CpG loci and 2,932 non-CpG cytosines, spanning gene-associated CpGs and a large number of enhancer/regulatory CpGs in intergenic regions^67^. Briefly, DNA was quantified by Qubit fluorimetry (Life Technologies), and 500 ng of DNA from each sample was bisulfite converted using the Zymo EZ DNA methylation kit (Zymo Research) following the manufacturer’s protocol using the specified modifications for the Illumina Infinium methylation assay. After conversion, all bisulfite reactions were cleaned using the Zymo-Spin binding columns and eluted in Tris buffer. Following elution, bisulfate-converted DNA was processed through the EPIC array protocol. For FFPE samples, the entire bisulfate-converted eluate was used as input for the Infinium HD FFPE DNA Restore kit and processed through the separate restoration workflow. To perform the assay, converted DNA was denatured with NaOH, amplified and hybridized to the EPIC bead chip. An extension reaction was performed using fluorophore-labeled nucleotides per the manufacturer’s protocol.

### DNA methylation data packages

DNA methylation data were packaged into the following four levels.

Level 1. Level 1 data contain raw IDAT files (two per sample with the extensions _Grn.idat and _Red.idat for the two-color channels) as produced by the Illumina iScan system. The mapping between IDAT file names and AURORA sample barcodes is provided in Sample.mapping.tsv.

Level 2. Level 2 data contain the signal intensities corresponding to methylated (M) and unmethylated (U) alleles and detection *P* values for each probe as extracted by the readIDATpair function in the R package SeSAMe (https://github.com/zwdzwd/sesame) from the IDAT files. The *P* values were calculated using pOOBAH (*P* value with out-of-band probes for array hybridization), which is based on empirical cumulative distribution function of the out-of-band signal from all type I probes^68^.

Level 3. Level 3 data contain *β* values defined as *S*_M_/(*S*_M_ + *S*_U_) for each locus calculated using the R package SeSAMe, where *S*_M_ and *S*_U_ represent signal intensities for methylated and unmethylated alleles. The raw signal intensities are first processed with background correction and dye bias correction. The background correction is based on the noob method^69^. The dye bias is corrected using a non-linear quantile interpolation-based method using the dyeBiasCorrTypeINorm function^68^; *β* values are then computed using the getBetas function. Probes with a detection *P* value greater than 0.05 in a given sample are masked as NA. Whether the probe is masked due to detection failure is recorded in an extra column (Masked_by_Detection_P_value) to distinguish from experiment-independent masking of probes (*N* = 105,454) subject to cross-hybridization and genetic polymorphism. The experiment-independent masking is based on the MASK_general column of the file named EPIC.hg38.manifest.tsv (release 20180909) downloaded from http://zwdzwd.github.io/InfiniumAnnotation^67^. From the same source, an additional file (EPIC.hg38.manifest.gencode.v22.tsv) is also included to provide detailed annotation of transcription association for each probe.

Level 4. Level 4 data contain a merged data matrix with *β* values across all samples. Probes masked as NA concerning the probe design in level 3 data were removed. Six FFPE samples that initially yielded low-quality data were rerun. The resulting two datasets values were merged probe-wise by taking the mean *β* value. If data were masked in one of the runs, we took available data from the other run.

#### Nomenclature for control samples

We included several cell line control samples in each batch to allow for the evaluation of potential batch effects and to facilitate correction of observed batch effects.

Control sample IDs that start with ‘VARI-Control-’ can be interpreted as

VARI-Control-[batch number]-[(cell line name)-(DNA isolate ID (A,B,..)]-[assay technical replicate (1,2,3…sequential across batches for the same DNA isolate)].

### External DNA methylation datasets

We processed additional normal tissue DNA methylation data from ENCODE and Gene Expression Omnibus (GEO). We collected raw IDAT files for 24 samples from seven tissue types, including adrenal gland (*n* = 5), liver (*n* = 1), lung (*n* = 4), ovary (*n* = 2), skin (*n* = 4), blood (*n* = 6) and brain (*n* = 2), that were frequently represented as a site of metastasis. We generated *β* values using the R package SeSAMe as described above for the AURORA samples. Further information on these datasets is provided in Supplementary Table 5.

### Global DNA hypermethylation analysis

To examine cancer-associated DNA hypermethylation profiles, we first used DNA methylation data from normal tissues to eliminate CpG sites that are involved in tissue-specific methylation (mean *β* value of >0.2 in any of the eight tissue types). We eliminated additional CpGs that were significantly differentially methylated between FF and FFPE samples (*t*-test FDR-adjusted *P* value of <0.01 and absolute mean *β* value difference of >0.25). For the heat map analysis shown in Fig. 1c, we used 5,000 of the most variably methylated CpGs across tumors. The probes lacked methylation in the normal tissues (*N* = 146,385), and the subset (*N* = 5,000) used in the heat map is listed in Supplementary Table 5. Tumor samples in the heat map in Fig. 1c were logically sorted as follows to help assess the similarity of DNA methylation profiles among matched samples: (1) cases were stratified by PAM50 call in the primary tumor; (2) within subtypes, cases were ordered by decreasing median *β* value in the primary tumor; (3) within cases, a primary tumor was listed first, followed by metastases for each case; and (4) metastases from the same case were ordered by decreasing tumor purity.

### Distal element DNA hypomethylation associated with metastasis

We identified 152,211 CpGs in distal enhancer-like signatures (dELSs), which fall more than 2 kilobases (kb) from the nearest transcription start site, defined by the ENCODE project^37^. We then selected 19,607 CpGs that are constitutively methylated across eight normal tissue types (mean *β* value of >0.8). Using the 19,607 CpGs sites, we fitted a probe-wise linear mixed-effects model with terms including primary versus metastasis, tumor purity and participant (coded as a random effect) as implemented in the R package lme4 (ref. 59). *P* values were estimated based on Satterthwaite’s approximation method included in the lmerTest^60^ package in R and adjusted for multiple testing using the Benjamini–Hochberg approach^61^. To examine transcription factors that bind to the CpG sites hypomethylated in metastatic tumors, we analyzed 11,348 ChIP–seq datasets on 1,359 individual DNA binding factors curated in the Cistrome Data Browser^70^. The statistical significance of enrichment for transcription factor binding sites among the hypomethylated CpGs was determined using Fisher’s exact test, with 200-bp regions centered on the target CpGs using the R package LOLA. All CpGs on the array overlapping the distal enhancer-like signatures were used as the background set. *P* values were adjusted for multiple comparisons using the Benjamini–Hochberg method^61^.

### Putative ESR1 and FOXA1 enhancer target genes affected by metastasis-associated DNA hypomethylation

We identified 47 significantly hypomethylated CpGs overlapping the binding sites for ESR1 or FOXA1. To investigate putative target genes affected by DNA hypomethylation, we first collected 4,681 putative targets of either ESR1 or FOXA1 in BCs as predicted by Cistrome Cancer^70^. We then considered at most 10 of the nearest genes within 1,000 kb upstream and 10 of the nearest genes within 1,000 kb downstream from the affected CpG sites, resulting in a list of 121 potential target genes (Supplementary Table 5). GO term overrepresentation analysis was performed using the enrichGO function with default parameters as implemented in the R package clusterProfiler.

### Identification of DNA hypermethylation associated with metastasis

To identify CpG sites hypermethylated in metastatic tumors compared to in primary tumors, we used 146,385 probes unmethylated in normal tissues defined above. We fitted a probe-wise linear mixed-effects model with terms including primary versus metastasis, tumor purity and participant (coded as a random effect) as implemented in the R package lme4 (ref. 59). *P* values were estimated based on Satterthwaite’s approximation method included in the lmerTest^60^ package in R and adjusted for multiple testing using the Benjamini–Hochberg approach^61^.

### CpG target analysis

Probes located in the PcG target sites (Fig. 6e,j,o) were determined using H3K27me3 ChIP–seq peaks on the H1 embryonic stem cells generated by the NIH Roadmap Epigenomics Consortium^71^. The broad peaks were downloaded using the R package AnnotationHub (ID AH28888).

### TCGA DNA methylation data

We analyzed the BC dataset from TCGA project, including 761 primary tumors and 74 adjacent non-malignant tissues profiled using the Infinium HumanMethylation450 (HM450) array (Supplementary Table 7). IDAT files were processed using the openSeSAMe pipeline implemented in the R package SeSAMe.

### DNA sequencing of tumor and normal tissue

Due to variable DNA quality, ranging from high (>2 kb; 131 samples) to medium (0.5–2 kb; 18 samples) and low (<0.5 kb; 44 samples), the 193 AURORA samples were binned into three different batches. For each batch, library construction used the NEBNext UltraII FS DNA library prep kit (New England Biolabs) with a 15-min enzymatic fragmentation. Each library received a unique dual-indexed adapter (Integrated DNA Technologies), allowing for both low-pass WGS and multiplex hybrid capture enrichment. Libraries were pooled at 2–4 µl based on final library quality and yield. To evaluate library representation due to variable DNA quality, we performed a survey of WGS sequencing for proper library balancing. The pooled libraries were concentrated and diluted to 2.25 nM for survey sequencing on the NovaSeq 6000.

Exome hybrid capture used the IDT xGen Exome Research Panel v1.0 enhanced with the xGenCNV Backbone Panel-Tech Access (Integrated DNA Technologies). The remaining pooled libraries were hybridized to this probe set according to the manufacturer’s protocol. The captured products were eluted following precipitation with streptavidin-labeled magnetic beads, amplified by PCR and quantitated before dilution and preparatory flow cell amplification for Illumina sequencing. Illumina paired-end sequencing (recipe: 151 × 17 × 8 × 151) was performed on the NovaSeq 6000 using the S4 flow cell configuration. For WGS, we targeted 5× coverage, and for whole-exome sequencing, we aimed for an average unique, on-target sequencing coverage depth of 500× for the tumor and 250× for the matched normal tissue.

### Churchill secondary analysis for DNA sequencing

The Nationwide Children’s Hospital (NCH)-developed Churchill secondary analysis pipeline^3^ was used to process paired-end read data for all samples, using attached unique molecular identifiers. Reads were aligned to reference genome GRCh38.d1.vd1 via bwa-mem, with the resulting alignment deduplicated using GATK’s (Picard) MarkDuplicates and base scores recalibrated using GATK’s BaseRecalibrator and ApplyBQSR. Variant calling was then performed on the final deduplicated, recalibrated BAM files. Germline variants were called using GATK’s HaplotypeCaller; somatic variants were called using GATK’s Mutect2, with the paired normal samples used to exclude germline variants. Somatic variant filters from Mutect2 were applied, and additional filtering of somatic variants from FFPE sources was performed using corrected variant allele frequency, read start diversity and unique read ends as indicators of preservation-sourced artifacts. Descriptions of the specific filters can be found below. All single-nucleotide variants (SNVs) and insertions and deletions (indels) were annotated via SnpEff using the GDC.h38 GENCODE v22 database. To ensure that samples were of usable quality, depth and breadth metrics were generated by mosdepth, oxidation and insert size metrics were generated by GATK’s CollectOxoGMetrics and CollectMultipleMetrics tools, and sequence usability (duplicate, softclipping, mapq0, unaligned) metrics were generated via samtools and custom scripts.

### FFPE filtering

#### FFPE_filter_LMR_VAF_0.04

Local mismatch rate-corrected variant allele frequency below 4%. The local mismatch rate of a variant is the number of mismatched bases in all reads aligned within a 10-bp window on each side of the position divided by the total number of bases aligned in this region. This value (LMR) is subtracted from the variant allele frequency, and if the result is below 4%, the variant will be filtered.

#### FFPE_filter_RSD

Read start diversity filter. The number of unique start positions of all variants supporting reads are counted (after soft trimming). For variants with over 15 supporting reads, at least four unique starting positions are required to pass this filter. For variants with over five supporting reads, at least two unique starting positions are required.

#### FFPE_filter_URE

Unique nearest read end filter. For all variant supporting reads, either the start position or the end position, whichever is closest to the variant (after soft trimming), is recorded. For variants with over 15 supporting reads, at least four unique positions are required to pass this filter. For variants with over five supporting reads, at least two unique positions are required.

### Analysis of genomic alterations between primary and metastatic tumors

For the analysis of significantly mutated genes between primary and metastatic tumors, we first filtered the MAF file to only include the following variant classifications: Frame_Shift_Del, Frame_Shift_Ins, In_Frame_Del, In_Frame_Ins, Missense_Mutation, Nonsense_Mutation, Nonstop_Mutation, Splice_Site and Translation_Start_Site. We next constructed a binary gene by sample matrix (1 = any mutation, 0 = no mutation) only using gene mutations that were present in 10 or more AURORA samples (*n* = 100). To mitigate the possible impact of FFPE artifacts coming mainly from primary tumors, mutation calls were filtered by removing any primary mutation calls that were not present in a paired metastatic sample (two primary samples without a paired metastatic sample were removed), with a total of 78 samples (39 pairs). Metastatic samples were aggregated by participant and were considered mutated if at least one metastatic sample for the participant was mutated. We constructed a contingency table for mutated or non-mutated samples and tested for statistical significance between primary tumor and metastasis using Fisher’s exact test.

### DNA copy number variations (CNVs) and LOH

Copy number changes and LOH events in WGS samples were detected using GATK’s GermlineCNVCaller, with the Churchill pipeline’s final BAM alignments as input. Reads were counted for CNV detection across a binned 1,000-nucleotide intervals, and allele counting for LOH detection was confined to single-nucleotide polymorphisms within gnomAD that had a frequency of 0.01% or greater. Germline CNV events were identified by comparing individual normal samples to a panel of normal samples composed of all other germline normal samples. Somatic CNV events were identified by comparing each somatic sample for an individual to that individual’s paired germline normal sample. Following this, CNV events were annotated with the symbols of genes they affected, producing gene-specific denoised log_2_ copy ratios.

Additionally, copy numbers derived from the raw denoised copy ratio signal were produced and plotted across the *HLA* locus chromosome 6:28510120–33480577. A smoothing factor was applied by reducing the number of regions into bins by 50-fold and calculating the mean log_2_ value for each bin. *HLA-A*/*HLA-B*/*HLA-C*/*HLA-DRB5* genes were specifically noted for overlap with prominent deletions in the region (log_2_ ratio < −0.75, focal mean difference between tumor and normal of >0.25 and ~40 kb upstream of the *HLA-A* gene). Following the same threshold applied to the *HLA-A* gene, the *B2M* gene was adjusted by tumor purity (>–0.4).

### DNA copy number analysis (CNA) between primary tumors and metastases

For the analysis of DNA copy number between primary tumors and metastases, we first collapsed the log_2_ copy ratio mean denoised values (gene-level CNA values) to 533 segment-level CNA scores. The complete list of genes in each segment was previously described^72^ (we excluded ‘Y chromosome’ and ‘chr2:53680282-53845245.BeroukhimS5.amp’ segments that scored 0 in all samples). Each segment score was calculated as the mean of gene-level CNA values across genes within the segment. CNA segment values were transformed into binary data (CN gain or loss cutoff of 0.2 and −0.2, respectively). Only samples in the WGS_DNA Seq FreezeSet 135 set and the Pairs_WGS_DNAseq sets of Supplementary Table 2 were used, and from the two primary samples for participant AER5, the A738_H04 sample was removed. We next compared CNA segment gains and losses in AURORA primary versus metastatic samples using Fisher’s exact test to determine if there were non-random associations between gain or loss on 46 primaries and 87 metastases. We constructed a contingency table for gains and a contingency table for losses for each segment of interest and tested for statistical significance.

### Clonality and tumor purity

Clonal variation within and among tumor samples was assessed using superFreq. Output BAM alignments from the Churchill pipeline were filtered down to only unique reads overlapping a probe-targeted region. The filtered alignments were then regenotyped using Varscan2 to identify the presence or absence of each of a case’s variants in each of its samples. With these inputs, superFreq assesses likely copy number and LOH events in combination with SNVs and indels to generate the most likely substructure of clones for each sample. The percent composition of tumor cells of all clones was totaled to determine the cellularity of each sample. For each clone, variants in ClinVar- and COSMIC-listed genes are highlighted as well as likely damaging mutations (frameshift and nonsense); these variants were then queried in the VarSome database, with ‘pathogenic’ and ‘likely pathogenic’ variants being considered as potentially consequential clonal variation..

### Neoantigen prediction

Somatic variants from samples where both RNAseq and DNA-sequencing data were available were evaluated as potential neoantigens using pVACseq, part of the pVACtools package. SNVs and indels, after Mutect2 filtering and FFPE filtering, when appropriate, were combined with gene expression data to identify and prioritize tumor-specific neoepitopes that are both expressed and have a predicted increased binding affinity compared to the wild-type epitope in the context of the participant’s *HLA* class I alleles. Parameters used within the pVACseq pipeline and subsequent filtering are included in Supplementary Table 6.

### Reporting summary

Further information on research design is available in the Nature Portfolio Reporting Summary linked to this article.

## Supplementary information

Reporting Summary

Supplementary Table 1 Supplementary Tables 1–8.

## Data Availability

Accession numbers and data sharing are summarized in Supplementary Table 7. Briefly, all newly generated data are in dbGAP (AURORA study: phs002622.v1.p1; RAP study: phs002429.v1.p1) and GEO (AURORA study: RNAseq data (GSE209998), DNA methylation data (GSE212375); RAP study: RNAseq data (GSE193103)). All of the resources used during the studies outlined in this manuscript are summarized in Supplementary Tables 1–5 and in the Methods. Supplementary Table 2 includes the clinical and molecular characteristics available for each cohort used in this manuscript. Previously published GEICAM trial data that were reanalyzed here are available in dbGAP (phs001866) and GEO (GSE147322). The human BC data were derived from the TCGA Research Network (http://cancergenome.nih.gov/). Previously published human TCGA-BRCA DNA methylation and TCGA-BRCA RNAseq data are available at NCI GDC (https://portal.gdc.cancer.gov/legacy-archive) and at dbGaP (phs000178) (https://gdac.broadinstitute.org/runs/stddata_latest/data/BRCA/20160128/), respectively. All other data supporting the findings of this study are available from the corresponding author upon reasonable request.

## Code availability

R packages and scripts used to analyze the data, along with input data, are explained in the Methods. All packages are public and are freely available online. No new code or mathematical algorithms were generated from this manuscript.
