# Supplementary material for: Multiomics in primary and metastatic breast tumors from the AURORA US network finds microenvironment and epigenetic drivers of metastasis
Source: Nat Cancer. 2022 Dec 30;4(1):128–47. doi: 10.1038/s43018-022-00491-x (PMC9886551; doi:10.1038/s43018-022-00491-x)
Supplement: Supplementary file 1 — Reporting Summary [file 43018_2022_491_MOESM1_ESM.pdf]

Corresponding author(s): Charles M. Perou
Last updated by author(s): Nov 3, 2022

Reporting Summary

Nature Portfolio wishes to improve the reproducibility of the work that we publish. This form provides structure for consistency and transparency in reporting. For further information on Nature Portfolio policies, see our Editorial Policies and the Editorial Policy Checklist.

Statistics

For all statistical analyses, confirm that the following items are present in the figure legend, table legend, main text, or Methods section.

n/a	Confirmed	
☐	☒	The exact sample size (*n*) for each experimental group/condition, given as a discrete number and unit of measurement
☐	☒	A statement on whether measurements were taken from distinct samples or whether the same sample was measured repeatedly
☐	☒	The statistical test(s) used AND whether they are one- or two-sided<br>*Only common tests should be described solely by name; describe more complex techniques in the Methods section.*
☐	☒	A description of all covariates tested
☐	☒	A description of any assumptions or corrections, such as tests of normality and adjustment for multiple comparisons
☐	☒	A full description of the statistical parameters including central tendency (e.g. means) or other basic estimates (e.g. regression coefficient) AND variation (e.g. standard deviation) or associated estimates of uncertainty (e.g. confidence intervals)
☐	☒	For null hypothesis testing, the test statistic (e.g. *F*, *t*, *r*) with confidence intervals, effect sizes, degrees of freedom and *P* value noted<br>*Give P values as exact values whenever suitable.*
☒	☐	For Bayesian analysis, information on the choice of priors and Markov chain Monte Carlo settings
☐	☒	For hierarchical and complex designs, identification of the appropriate level for tests and full reporting of outcomes
☐	☒	Estimates of effect sizes (e.g. Cohen's *d*, Pearson's *r*), indicating how they were calculated

Our web collection on statistics for biologists contains articles on many of the points above.

Software and code

Policy information about availability of computer code

Data collection	We collected 55 patients (51 primary tumors and 102 metastatic samples). All data collected during this study has been included in the results section "Clinical features of the cohort and global genomic patterns" and mainly in the Methods section. Tumor DNA and RNA were isolated from each specimen and utilized, assuming sufficient quality and quantity, in four different genomic assays: DNA exomes and low-pass whole genome sequencing of tumor and normal (WES/WGS), whole transcriptome RNAseq (RNAseq) using rRNA depletion, and DNA methylation microarrays (DNAme). In total, we sequenced 134 tumors with WES, 131 tumors with WGS, 123 tumors with RNAseq and 131 tumors with DNAme assays. 87/153 specimens had all 4 assays successfully performed. More details about the kits, technology and pipelines used for quantification and processing of RNA/DNA sequencing and DNA methylation microarrays are included in the methodology section.
Data analysis	Clinical, RNAseq, DNAseq and DNAme analysis was performed using RStudio version 1.4.1103 (http://cran.r-project.org), GraphPad Prism® 9.0 software and/or Microsoft Excel® (for Microsoft 365 MSO (Version 2210 Build 16.0.15726.20070). More details about each particular platform analysis are found on the Methodology section.<br>R packages and scripts used to analyze the data, along with input data, are explained in the methodology section. All packages are public and are freely available online. No new codes or mathematical algorithms were generated from this manuscript.

For manuscripts utilizing custom algorithms or software that are central to the research but not yet described in published literature, software must be made available to editors and reviewers. We strongly encourage code deposition in a community repository (e.g. GitHub). See the Nature Portfolio guidelines for submitting code & software for further information.

Data

Policy information about availability of data

All manuscripts must include a data availability statement. This statement should provide the following information, where applicable:

- Accession codes, unique identifiers, or web links for publicly available datasets
- A description of any restrictions on data availability
- For clinical datasets or third party data, please ensure that the statement adheres to our policy

Accession numbers and data sharing: All newly generated data is in dbGAP (AURORA study: phs002622.v1.p1; RAP study: phs002429.v1.p1) and GEO (AURORA study: RNASeq data (GSE209998), DNA Methylation data (GSE212375); RAP study: RNAseq data (GSE193103). All of the resources used during this manuscript are summarized in Supplementary table 1-5 and in the methodology section. Supplementary table 2 includes the clinical and molecular characteristics available for each cohort used in this manuscript. Previously published GEICAM/2009-03 ConvertHER trial data that were re-analyzed here are available in dbGAP (phs001866) and GEO (GSE147322). The human breast cancer data were derived from the TCGA Research Network: http://cancergenome.nih.gov/. Previously published human TCGA-BRCA DNA methylation and TCGA-BRCA RNAseq data are available at NCI GDC https://portal.gdc.cancer.gov/legacy-archive) and at dbGaP (accession phs000178) (https://gdac.broadinstitute.org/runs/stddatalatest/data/BRCA/20160128/), respectively.
Source data have been provided as Source Data files. All other data supporting the findings of this study are available from the corresponding author upon reasonable request.

Human research participants

Policy information about studies involving human research participants and Sex and Gender in Research.

Reporting on sex and gender	All patients used in this study were females.
Population characteristics	Samples from a total of 55 patients with metastatic breast cancer were the final data set of the AURORA US cohort. Of these 55 women, 10 (18%) were of African American descent and 4 (7%) were of Hispanic ethnicity. Median age at initial breast cancer diagnosis was 49 years (range: 25-76). Forty-nine patients (89%) initially presented with stage I-III breast cancer, of which 19 (38%) received neoadjuvant systemic therapy, and six patients (10%) presented with de novo metastatic disease. Ductal histology was most prevalent among the cohort (n=44, 80%); 7 patients (12%) were diagnosed with lobular or mixed lobular/ductal carcinoma. The distribution of breast cancer receptor subtype per clinical testing at initial diagnosis was triple-negative, n=19 (34%); hormone receptor (HR)-positive/HER2-negative, n=17 (30%); HR-positive/HER2-positive, n=6 (10%); HR-negative/HER2-positive, n=4 (7%); and unknown, n=9 (16%). In the metastatic setting, patients received a median of 3 lines of systemic therapy (range: 0-20). Metastatic samples from a total of 20 patients were collected at autopsy. Additional clinicopathologic features are displayed in Table 1 and Supplementary Table 1 of the manuscript.
Recruitment	Each participating institution provided samples from existing banked tissues with appropriate permissions for secondary research use. All de-identified patient clinical data was collected in a central RedCap database (https://projectredcap.org/software/).
Ethics oversight	This research complies with all relevant ethical regulations and was approved by Institutional Review Boards and Offices of Research at Baylor College of Medicine, Dana Farber Cancer Institute, Duke University, Georgetown University Medical Center, Indiana University, Mayo Clinic, Memorial Sloan Kettering Cancer Center, University of Pittsburgh, and University of North Carolina at Chapel Hill.

Note that full information on the approval of the study protocol must also be provided in the manuscript.

Field-specific reporting

Please select the one below that is the best fit for your research. If you are not sure, read the appropriate sections before making your selection.

☒ Life sciences ☐ Behavioural & social sciences ☐ Ecological, evolutionary & environmental sciences

For a reference copy of the document with all sections, see nature.com/documents/nr-reporting-summary-flat.pdf

Life sciences study design

All studies must disclose on these points even when the disclosure is negative.

Sample size	Sample size was limited by the size of the samples provided and successfully assayed for this study
Data exclusions	No data was excluded from the analysis
Replication	Replicates are indicated in each figure and corresponding figure legend.

Randomization	Randomization was not applicable to the study
Blinding	Blinding was not applicable to the study

Reporting for specific materials, systems and methods

We require information from authors about some types of materials, experimental systems and methods used in many studies. Here, indicate whether each material, system or method listed is relevant to your study. If you are not sure if a list item applies to your research, read the appropriate section before selecting a response.

Materials & experimental systems

n/a	Involved in the study	
☐	☒	Antibodies
☒	☐	Eukaryotic cell lines
☒	☐	Palaeontology and archaeology
☒	☐	Animals and other organisms
☒	☐	Clinical data
☒	☐	Dual use research of concern

Methods

n/a	Involved in the study	
☒	☐	ChIP-seq
☒	☐	Flow cytometry
☒	☐	MRI-based neuroimaging

Antibodies

Antibodies used	PanCK AE1/AE3 Biocare, at 1:1600; HLA-A C6 Santa Cruz at 1:1300.
Validation	AE1/AE3 s a mouse monoclonal that recognizes the acidic and basic (Type I and II) subfamilies of cytokeratins. The cocktail of these two antibodies can be used to detect most human epithelia. The acidic cytokeratins have molecular weights of 56.5, 55, 51, 50, 50, 48, 46, 45, and 40 kDa. The basic cytokeratins have molecular weights of 65-67, 64, 59, 58, 56 and 52 kDa. HLA-A (C-6) is a mouse monoclonal antibody specific for an epitope mapping between amino acids 61-93 within an internal region of HLA-A of human origin. Antibody testing was performed on control tissues with chromogenic and fluorescence immunohistochemistry (IHC) to ensure expression patterns corresponding to their biologically expected distribution. Tonsil and placenta were used as a positive and negative control tissues.<br>Clin Cancer Res. 2021 Oct 1;27(19):5299-5306. doi: 10.1158/1078-0432.CCR-21-0607
